# Factors associated with excess all-cause mortality in the first wave of the COVID-19 pandemic in the UK: A time series analysis using the Clinical Practice Research Datalink

**DOI:** 10.1371/journal.pmed.1003870

**Published:** 2022-01-06

**Authors:** Helen Strongman, Helena Carreira, Bianca L. De Stavola, Krishnan Bhaskaran, David A. Leon

**Affiliations:** 1 London School of Hygiene & Tropical Medicine, London, United Kingdom; 2 University College London, London, United Kingdom; 3 UiT The Arctic University of Norway, Tromsø, Norway; 4 National Research University Higher School of Economics, Moscow, Russia; Harvard Medical School, UNITED STATES

## Abstract

**Background:**

Excess mortality captures the total effect of the Coronavirus Disease 2019 (COVID-19) pandemic on mortality and is not affected by misspecification of cause of death. We aimed to describe how health and demographic factors were associated with excess mortality during, compared to before, the pandemic.

**Methods and findings:**

We analysed a time series dataset including 9,635,613 adults (≥40 years old) registered at United Kingdom general practices contributing to the Clinical Practice Research Datalink. We extracted weekly numbers of deaths and numbers at risk between March 2015 and July 2020, stratified by individual-level factors. Excess mortality during Wave 1 of the UK pandemic (5 March to 27 May 2020) compared to the prepandemic period was estimated using seasonally adjusted negative binomial regression models. Relative rates (RRs) of death for a range of factors were estimated before and during Wave 1 by including interaction terms. We found that all-cause mortality increased by 43% (95% CI 40% to 47%) during Wave 1 compared with prepandemic. Changes to the RR of death associated with most sociodemographic and clinical characteristics were small during Wave 1 compared with prepandemic. However, the mortality RR associated with dementia markedly increased (RR for dementia versus no dementia prepandemic: 3.5, 95% CI 3.4 to 3.5; RR during Wave 1: 5.1, 4.9 to 5.3); a similar pattern was seen for learning disabilities (RR prepandemic: 3.6, 3.4 to 3.5; during Wave 1: 4.8, 4.4 to 5.3), for black or South Asian ethnicity compared to white, and for London compared to other regions. Relative risks for morbidities were stable in multiple sensitivity analyses. However, a limitation of the study is that we cannot assume that the risks observed during Wave 1 would apply to other waves due to changes in population behaviour, virus transmission, and risk perception.

**Conclusions:**

The first wave of the UK COVID-19 pandemic appeared to amplify baseline mortality risk to approximately the same relative degree for most population subgroups. However, disproportionate increases in mortality were seen for those with dementia, learning disabilities, non-white ethnicity, or living in London.

## Abstract 2

Helen Strongman and colleagues investigate the health and demographic factors associated with excess mortality during, as compared to before, the COVID-19 pandemic.

## Author summary

**Why was this study done?:**

- All-cause mortality during the Coronavirus Disease 2019 (COVID-19) pandemic was substantially higher than in previous years, particularly among elderly people, males, people of non-white ethnicity, people of lower socioeconomic status, and people living in care homes.
- There is little evidence of how preexisting health and demographic factors are associated with all-cause mortality and how this compares to mortality risks in people with and without these factors before the pandemic.

**What did the researchers do and find?:**

- We used seasonally adjusted time series models to estimate all-cause and excess mortality during Wave 1 of the UK pandemic (5 March to 27 May 2020) for people with individual health and sociodemographic factors; we then estimated relative risks of all-cause mortality in people with and without these factors before the pandemic and during Wave 1.
- We found similar relative risks of all-cause mortality in people with and without each factor before and during Wave 1 of the pandemic, but there were exceptions.
- Health-related exceptions were people with a diagnosis of dementia or learning disabilities, whose risk of death relative to people without these conditions was substantially higher during Wave 1 than it was before the pandemic.
- Socioeconomic exceptions were non-white ethnicity and living in London; these groups had lower immediate risks of death before the pandemic compared to white people or people living outside of London, but substantially elevated relative risks during Wave 1.

**What do these findings mean?:**

- This suggests that COVID-19 has dialled up the risk of death by a similar proportional degree for most people.
- From a clinical perspective, prepandemic knowledge about the relative frailty associated with different conditions can be reasonably applied in the pandemic situation, though further follow-up will be needed to confirm that our observations generalise beyond Wave 1.
- Exceptions include people with learning difficulties and dementia who may have been more exposed to infection in care homes and through personal care and those of non-white ethnicity who may have been at higher risk due to increased social interactions.

## Introduction

Understanding how preexisting health status and demographic factors affect the risk of infection and death from Coronavirus Disease 2019 (COVID-19) has been a critical clinical and public health priority since the start of the pandemic. This knowledge informs optimal care and treatment of those infected with Severe Acute Respiratory Syndrome Coronavirus 2 (SARS-CoV-2) as well as policies on shielding and vaccination. A number of studies of COVID-19 mortality in relation to health and demographic factors have highlighted factors such as age, deprivation, ethnicity, and diabetes as particular risk factors for death from COVID-19 [1–9]. Studies of the impact of health and demographic factors on mortality from conditions other than COVID-19 during the pandemic have been relatively uncommon. An analysis of over 17 million adults registered with primary care practices in England [10] found that most factors associated with COVID-19 death were similarly associated with non-COVID death. A key limitation of this study is reliance on cause-specific mortality data. In the United Kingdom, in the first months of the 2020 pandemic, it is very likely that attribution of deaths to COVID-19 was unreliable because of the novelty of the virus, uncertainty about its precise clinical manifestations, and an absence of widespread testing for laboratory confirmation of infection. The problem of attribution of cause is further complicated by the probability that some deaths may have occurred as a result of indirect effects, such as reduced diagnosis and care for non-COVID conditions, plus the broader effect of voluntary and obligatory behavioural change such as social distancing.

One approach to circumventing the challenge of analysing cause-specific mortality has been to assess the impact of the pandemic on total excess deaths [11], a methodology that compares the number of deaths from any cause during the pandemic with the number expected in the same period based on mortality rates in earlier (prepandemic) years; this has been previously used to study the impact of seasonal influenza and acute exposures such as heat waves. Total excess mortality captures both the direct and indirect impacts of COVID-19 on mortality and is not affected by validity of cause of death. Excess mortality rates during the pandemic have been estimated for national and subnational populations [12–16]. de Lusignan and colleagues compared mortality in patients registered in a sample of general practices during Wave 1 with external life table data from 2018 to show that excess mortality may be particularly raised in men, older individuals, black people, and those with certain comorbidities; key factors such as dementia were not studied [17]. To our knowledge, there are no published analyses covering a wide range of health and demographic factors and comparing mortality rates prepandemic with those in the pandemic period within the same study population. Observational studies have therefore assumed that relative excess mortality in people with and without comorbidities was the same during and prior to the pandemic [17,18].

We estimated excess mortality in the UK during Wave 1 of the COVID-19 pandemic in people with an extensive range of morbidities (including dementia) and sociodemographic characteristics. We then estimated and compared relative risks of all-cause mortality in people with and without these factors before the pandemic and during Wave 1. To do this, we used a large regularly updated primary care database covering 20% of the UK population.

## Methods

### Study design and setting

We conducted a population-based time series study using data prospectively collected from 5 March 2015 to 31 July 2020 from the UK Clinical Practice Research Datalink (CPRD). The predefined study protocol is available in S1 Text. The study is reported as per the Reporting of studies Conducted using Observational Routinely-collected health Data (RECORD) Statement ([19]; S2 Text), which is an extension of the Strengthening the Reporting of Observational Studies in Epidemiology (STROBE) guideline.

General practices have an important role in the UK’s National Health Services, with responsibility for primary care and specialist referrals for the vast majority of the UK population [20]. General practitioners (GPs) record clinical diagnoses, hospital diagnoses that affect patients’ ongoing care, primary care prescribing, test, and laboratory data, in specialised software systems. Clinical diagnoses are recorded using Read or Snomed codes. In our primary analyses, we used data from CPRD GOLD [21] and CPRD Aurum [22], which include anonymised data collected from 2 software systems. Individually, these databases are broadly representative of the UK population in terms of age and sex [21,22]. Together, they cover over 20% of the UK population, distributed across all UK countries and English regions.

CPRD primary care data may be linked to additional health and area-based deprivation datasets. Quintiles of the Carstairs Index and urban–rural data were added by CPRD through linkage using the practice postcode [23]. Linkages based on patient data are also available [24] but are restricted to English practices that have agreed to participate in the linkage programme, reducing geographic coverage and the sample size. In sensitivity analyses, we used the Office for National Statistics (ONS) mortality data (national death registration data), ICD-10 coded clinical data from Hospital Episode Statistics Admitted Patient Care (HES APC) [25], and patient-level quintiles of Carstairs Index.

### Study population and procedures

We constructed a time series dataset from a study population that included all adults (≥40 years old) actively registered during the study period in a general practice contributing to CPRD on 31 July 2020. Follow-up in the base population started at the latest of 5 March 2015, 40th birthday, and 1 year after registration with the practice. Follow-up ended at the earliest of the end of the study period (i.e., 31 July 2020), the date the patient left the practice or died. Birth dates were estimated at 1 July of the year of birth as the day and month are not collected by CPRD. Date of death was identified from the CPRD-derived death date, which uses data recorded in general practices and has been shown to agree closely with the ONS-registered death date [26].

Number of deaths and number at risk were counted for each week of the year from 5 March 2015 to 31 July 2020. Each week was classified as “prepandemic” (before 5 March 2020), during Wave 1 of the pandemic (5 March to 27 May 2020), and after Wave 1 (28 May to 31 July).

Demographic factors included 5-year age groups, sexes, deprivation quintiles, ethnicities, and regions. Health factors included body mass index (BMI) categories, smoking status, and morbidities. Weekly numbers of deaths and numbers at risk were obtained separately for people with each health and demographic factor. For all morbidities except asthma and cancer, individuals were included in the exposed numbers at risk (of death) if they had a record of that morbidity prior to the start of the week. Asthma counts required a record in the last 3 years; for cancer, the first ever record was required to be in the past year. Weekly numbers of deaths and numbers at risk for people without each morbidity (the unexposed) were obtained by subtracting counts for each morbidity from the total study population counts. For BMI and smoking status, the most recent records prior to each week were used to define the category at the start of each week. For demographic factors other than age, categories were assigned at the start of follow-up. For descriptive purposes, counts were obtained for missing ethnicity, BMI, and smoking status.

A complete list of the factors included in the analyses is given in Table 1; full definitions are provided in S3 Text. Code lists for all study variables are available online (https://doi.org/10.17037/DATA.00002269).

**Table 1:** Number of person-weeks and observed, expected, and excess number of deaths during Wave 1 (5 March to 27 May 2020).

Stratifying factor	Person-weeks in millions (% weeks in base population)	Observed deaths (% deaths in study population)	Observed deaths per million person-weeks	Expected deaths per million person-weeks (95% CI)	Excess deaths per million person-weeks (95% CI)	
Study population	88.1 (100.0)	35,369 (100.0)	401	272 (271–272)	130 (129–130)	
**Age group**						
40 to 49	23.7 (26.9)	894 (2.5)	38	30 (30–30)	8 (8–8)	
50 to 59	24.4 (27.7)	2,262 (6.4)	93	66 (66–66)	26 (26–27)	
60 to 69	18.1 (20.6)	4,115 (11.6)	227	165 (164–165)	62 (62–63)	
70 to 79	13.9 (15.8)	8,253 (23.3)	593	411 (410–412)	181 (180–182)	
80 plus	7.9 (9.0)	19,845 (56.1)	2,504	1,619 (1,614–1,624)	885 (880–890)	
**Sex**						
Male	43.5 (49.3)	17,907 (50.6)	412	276 (275–277)	136 (136–137)	
Female	44.7 (50.7)	17,462 (49.4)	391	268 (267–268)	123 (123–124)	
**Deprivation** *						
1 (least deprived)	12.6 (14.3)	4,502 (12.7)	358	239 (239–240)	119 (118–120)	
2	17.5 (19.8)	6,764 (19.1)	387	264 (264–265)	123 (122–124)	
3	20.1 (22.8)	8,186 (23.1)	408	283 (282–284)	125 (125–126)	
4	18.9 (21.4)	7,922 (22.4)	420	290 (290–291)	129 (128–130)	
5 (most deprived)	17.5 (19.8)	7,416 (21.0)	425	268 (267–269)	157 (156–158)	
**Urban**–**Rural**^**$**^						
Urban	75.77 (86.0)	30,639 (86.6)	404	270 (270–271)	134 (133–135)	
Rural	12.60 (14.3)	4,865 (13.8)	386	283 (282–284)	103 (102–104)	
**Ethnicity**						
White	57.4 (65.1)	23,473 (66.4)	409	282 (281–283)	127 (127–128)	
South Asian	4.2 (4.7)	1,057 (3.0)	254	128 (127–129)	126 (125–127)	
Black	2.7 (3.0)	904 (2.6)	339	138 (137–139)	201 (200–203)	
Other and mixed	1.9 (2.2)	369 (1.0)	189	88 (87–89)	102 (101–103)	
Missing	22.2 (25.2)	9,701 (27.4)	437	307 (306–308)	130 (129–131)	
**Region**						
North East	2.35 (2.7)	1,194 (3.4)	507	325 (323–327)	183 (181–185)	
North West	13.30 (15.1)	5,910 (16.7)	444	299 (298–300)	146 (145–147)	
Yorkshire and the Humber	2.4 (2.7)	941 (2.7)	401	296 (295–298)	105 (103–107)	
East Midlands and East of England	4.5 (5.1)	1,630.0 (4.6)	365	250 (248–251)	115 (114–116)	
West Midlands	11.9 (13.5)	5,042 (14.3)	425	283 (282–284)	142 (141–143)	
South West	8.7 (9.9)	3,207 (9.1)	369	278 (277–279)	91 (90–92)	
South Central	9.2 (10.4)	3,451 (9.8)	377	254 (253–255)	123 (122–124)	
London	13.2 (15.0)	4,858 (13.7)	367	192 (192–193)	175 (174–175)	
South East Coast	7.6 (8.6)	2,964 (8.4)	389	262 (261–263)	128 (127–129)	
Northern Ireland	1.9 (2.2)	714 (2.0)	369	292 (290–294)	77 (75–79)	
Scotland	7.8 (8.8)	3,152 (8.9)	405	309 (308–310)	96 (95–97)	
Wales	5.4 (6.1)	2,306 (6.5)	429	327 (326–329)	102 (100–104)	
**Morbidity**						
**Autoimmune condition**						
Psoriasis	4.2 (4.8)	2,019 (5.7)	479	324 (323–326)	155 (153–156)	
Rheumatoid arthritis	1.3 (1.4)	1,100 (3.1)	872	588 (585–592)	284 (280–287)	
**Cardiovascular disease**						
Cerebrovascular disease	3.7 (4.2)	7,159 (20.2)	1,930	1,274 (1,270–1,278)	656 (652–660)	
Venous thromboembolism	2.6 (3.0)	3,844 (10.9)	1,453	1,016 (1,012–1,020)	437 (433–441)	
Chronic heart disease	7.5 (8.5)	11,885 (33.6)	1,590	1,113 (1,110–1,117)	477 (474–480)	
Hypertension	35.3 (40.1)	25,893 (73.2)	733	498 (497–499)	235 (234–237)	
**Chronic respiratory disease**						
Asthma	8.0 (9.1)	3,367 (9.5)	420	291 (290–293)		129 (128–130)
Other	5.2 (5.9)	7,111 (20.1)	1,376	1,040 (1,036–1,043)		336 (333–340)
**Neurological conditions associated with respiratory infection**						
Dementia	1.8 (2.0)	9,937 (28.1)	5,618	2,929 (2,918–2,941)		2,688 (2,677–2,699)
Learning disabilities	0.4 (0.5)	397 (1.1)	994	433 (427–438)		561 (556–567)
Other associated†	1.5 (1.7)	2,314 (6.5)	1,531	971 (967–976)		560 (555–564)
**Other morbidity**						
Chronic kidney disease	12.1 (13.7)	20,255 (57.3)	1,677	1,094 (1,091–1,097)		583 (580–586)
Cancer (diagnosed in last year)	0.6 (0.7)	2,740 (7.7)	4,275	3,659 (3,647–3,671)		616 (603–628)
Diabetes	9.8 (11.2)	9,878 (27.9)	1,003	643 (641–645)		361 (359–363)
Multimorbidity	3.5 (4.0)	6,780 (19.2)	1,929	1,398 (1,394–1,402)		531 (526–535)
**Health indicators**						
**BMI**						
<18.5 (Underweight)	1.3 (1.4)	3,144 (8.9)	2,465	1,830 (1,823–1,838)		635 (627–643)
18.5–<25 (Normal weight)	26.7 (30.3)	13,002 (36.8)	487	338 (337–339)		149 (148–150)
25–<30 (Overweight)	30.3 (34.4)	9,592 (27.1)	316	219 (218–219)		98 (97–98)
30–<35 (Obesity class I)	15.2 (17.2)	4,703 (13.3)	310	195 (194–196)		115 (114–115)
> = 35 (Obesity class II plus)	8.6 (9.8)	2,868.0 (8.1)	333	216 (215–217)		117 (116–118)
Missing	6.3 (7.1)	2,166 (6.1)	344	195 (194–196)		149 (148–150)
**Smoking status**						
Nonsmoker	28.1 (31.9)	8,400 (23.7)	299	187 (187–188)		111 (111–112)
Current smoker	9.7 (11.0)	3,539 (10.0)	367	305 (304–306)		61 (60–62)
Ex-smoker	48.3 (54.8)	22,678 (64.1)	470	318 (317–319)		152 (151–152)
Missing	2.3 (2.6)	863 (2.4)	377	200 (199–202)		177 (175–178)

*Carstairs Index. This in not available in Northern Ireland.

^$^Two mixed Urban–Rural practices in Northern Ireland were reclassified as Urban.

†associated with respiratory infections

For time-updating stratifying factors (e.g., BMI and smoking status), the sums of person-weeks in millions and observed deaths do not equal the study population. This is because periods of person time were counted from the first full week of follow-up. Short periods of person time from the date the stratifying factor changed to the start of the following week were not therefore counted.

BMI, body mass index.

### Statistical analysis

We fitted generalised linear models with a negative binomial error structure to the weekly counts of deaths, taking account of the numbers at risk in each week (the denominator sizes [27], by offsetting it after log transformation). All models included 3 pairs of Fourier terms (for creating a harmonic function of calendar week) to capture seasonality in death rate; 3 pairs of terms were chosen as we wished to adequately capture some degree of complexity in seasonal patterns without overfitting. A quadratic function of calendar year (treated as a continuous variable) was used to capture any major underlying trends allowing for nonlinearity [28]. We refer to this as the basic model. The estimation equation for the basic model is included in S4 Text.

#### Overall and relative excess mortality

The basic model was fitted using only the data from the prepandemic period, initially for the overall population and then restricted to each factor in turn (e.g., black ethnicity, overweight, dementia, and diabetes), to predict the number of deaths to be expected during the pandemic period (during and after Wave 1). We checked the adequacy of our generalised linear models by comparing observed and expected deaths in the prepandemic period; we additionally assessed the distribution of the deviance residuals and estimated mean weekly deviance residuals as a proportion of weekly death counts. We calculated excess deaths during Wave 1 of the pandemic by subtracting total predicted deaths during this period from total observed deaths. We estimated 95% confidence intervals by pooling the weekly standard errors for predicted deaths [29].

#### Association between individual factors and mortality before and during Wave 1

We estimated relative rates (RRs) of death for each health and demographic factor in separate models, allowing for an interaction with the time period (before versus during Wave 1 of the pandemic) and adjusting for age and sex differences. This was done by extending the basic model to include a binary pandemic indicator (1 for Wave 1, 0 otherwise), the binary/categorical variable representing the health or demographic factor grouping (e.g., diabetes, ethnicity, and BMI), and terms capturing their interaction. Age (in 5-year age groups) and sex were also included in each model to adjust for differences in age and sex structure by health or sociodemographic status (e.g., people with or without diabetes and black ethnicity versus white ethnicity). Missing categories for ethnicity, BMI, and smoking status were excluded in these primary analyses. We examined partial autocorrelation plots and found no evidence of residual autocorrelation in the final models [28].

#### Secondary and sensitivity analyses

In secondary analyses, we described differences between age groups and sex. For cancer, previous evidence shows an increased risk of poor COVID-19 outcomes in haematological cancer patients [1], and thus we also estimated RRs of death separately for haematological and nonhaematological cancer patients, all diagnosed in the last year. As people recently diagnosed with cancer may have been more motivated to avoid infection than long-term cancer survivors, we further extended our analyses to include people who had cancer diagnosed >1 year and <5 years ago and more than 5 years ago.

To examine the robustness of our findings, we completed sensitivity analyses using additional linked data. The study population was restricted to individuals registered in English practices who were included in CPRD’s linkage programme [24]. The ONS mortality date was used in place of the CPRD derived death date, HES data were used in addition to primary care data to define ethnicity and morbidities (except asthma), and the patient postcode level Carstairs Index was used to define deprivation. Additional sensitivity analyses compared findings using the CPRD GOLD and Aurum databases and the addition of a missing data category to the RR analyses for ethnicity, BMI, and smoking status.

We used StataMP 16.0 for all analyses. All analytical code is available at https://doi.org/10.17037/DATA.00002269.

### Ethics statement

This study was approved by the London School of Hygiene & Tropical Medicine Ethics Committee (22680) and the Independent Scientific Advisory Committee for the Medicines and Healthcare products Regulatory Agency database research (20_163R). CPRD supplies anonymised data for public health research; therefore, individual patient consent was not required for this study.

## Results

**Fig 1:**
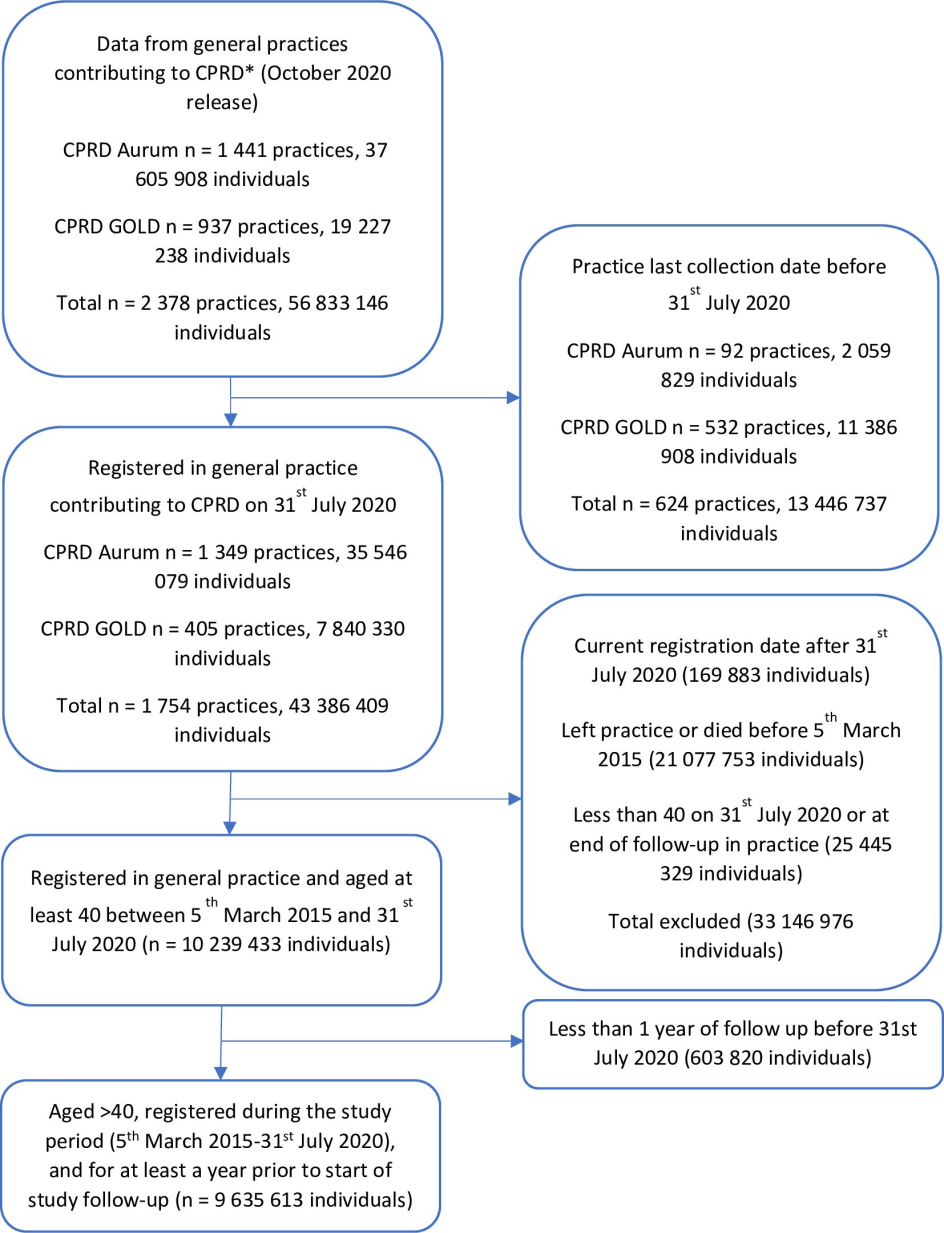
Study cohort selection for primary analysis. *Practices may contribute to both CPRD GOLD and CPRD Aurum over different time periods. Initial cohort restricted to patients who were permanently registered in the practice with valid registration data (termed “acceptable” by CPRD). CPRD, Clinical Practice Research Datalink.

Our primary analysis was based on 9,635,613 individuals aged at least 40 with a minimum 1 year of follow-up and who were registered in 1,754 practices that contributed data to CPRD GOLD or Aurum on 31 July 2020 (Fig 1). Median follow-up in the base population was 5.4 years (IQR 2.3 to 5.4) with individuals contributing 1,989.8 million person-weeks of follow-up, 88.1 million person-weeks of which were during Wave 1. A total of 585,170 deaths were observed of which 35,369 were in Wave 1. The number of individuals contributing each week increased gradually over time (S1 Fig).

### Overall excess mortality and mortality rate ratio

**Fig 2:**
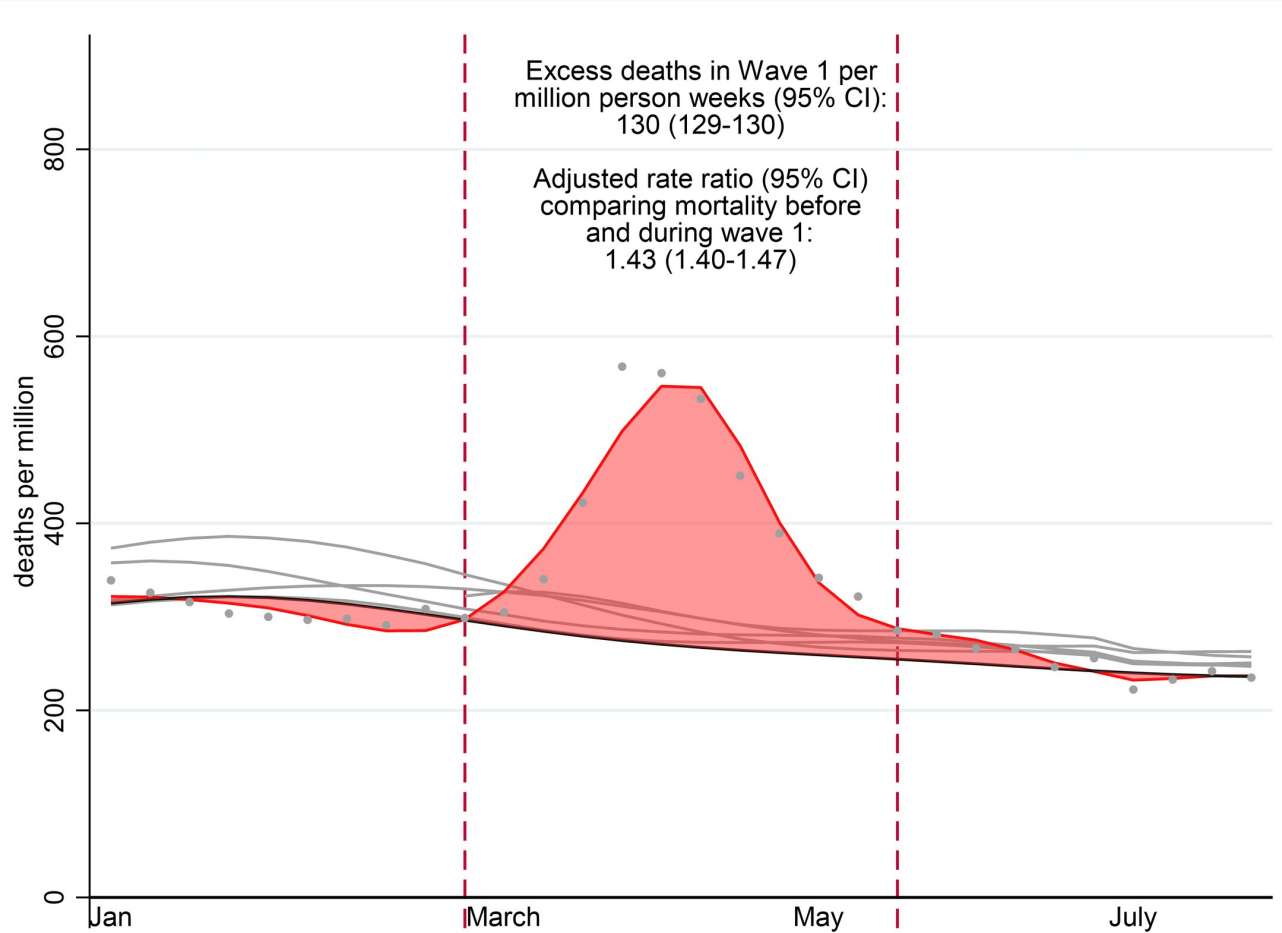
Observed number of deaths from all causes per million person-weeks in 2020 before and during the pandemic and year-specific fitted curves for the years 2015 to 2020. Solid grey lines: model fitted numbers of deaths in 2015–2019 (predicted from year and season model restricted to 2015 to 2019). Grey dots: Observed deaths in 2020. Solid red line: model fitted number of deaths in 2020 using natural cubic spline (with 8 knots per year before the pandemic and an additional 3 knots during the pandemic). Solid black line: predicted number of deaths in 2020 from year and season model fitted on 2015–2019 data. Dotted red lines: start and end of Wave 1. Red shaded area: excess number of deaths during the pandemic. Excess deaths per million patient-weeks = Total number of observed deaths (grey dots) in Wave 1 of the pandemic minus predicted number according to the 2015–2019 model (solid black line) Adjusted rate ratio of Wave 1 versus prepandemic period–Estimated by the model fitted on the full study population adjusted for age, sex, and annual and seasonal effects.

Fig 2 shows overall patterns of observed, expected, and excess mortality for the full study period. We observed 401 deaths per million person-weeks in Wave 1 compared with an estimated 272 (271 to 272), indicating that 130 (95% CI 129 to 130) excess deaths occurred per million person-weeks during this period. The RR of death in Wave 1 compared to the prepandemic period, adjusted for seasonality, year, age, and sex was 1.43 (95% CI 1.40 to 1.47). Patterns of observed and predicted deaths for the full study period for each comorbidity, taking annual and seasonal patterns into account, are described in S2 Fig; mean (SD) deviance residuals are tabulated in S1 Table. Model fit was good for most covariates; deviance residuals were near-normally distributed with few outliers. Mean absolute deviance as a proportion of weekly deaths was low for the study population (mean 0.04 SD 0.03) and highest for comorbidities with lower death counts such as learning difficulties (mean 0.24 SD 0.27). Seasonal patterns were consistent, except for people with recent cancer diagnoses where they were less pronounced. Mortality rates peaked in the severe 2017/2018 influenza season and decreased gradually in subsequent years.

### Excess mortality by health and demographic factors

Table 1 describes the population at risk, observed, expected, and excess deaths during Wave 1 of the pandemic for each factor. Most deaths occurred among the elderly population and in people who had preexisting chronic conditions, notably hypertension, chronic kidney disease, and dementia.

The number of excess deaths per million person-weeks varied greatly between factors. This is demonstrated by the 100-fold difference between those aged 40 to 49 and those aged 80 plus years. Numbers of excess deaths per million person-weeks were highest among people with dementia (2,693; 95% CI 2,682 to 2,704), cerebrovascular disease (656; 95% CI 652 to 660) or cancer diagnosed in the last year (616; 95% CI 603 to 628), and for people who were underweight (628; 95% CI 620 to 635).

### Mortality rate ratios by health and demographic factors

**Fig 3:**
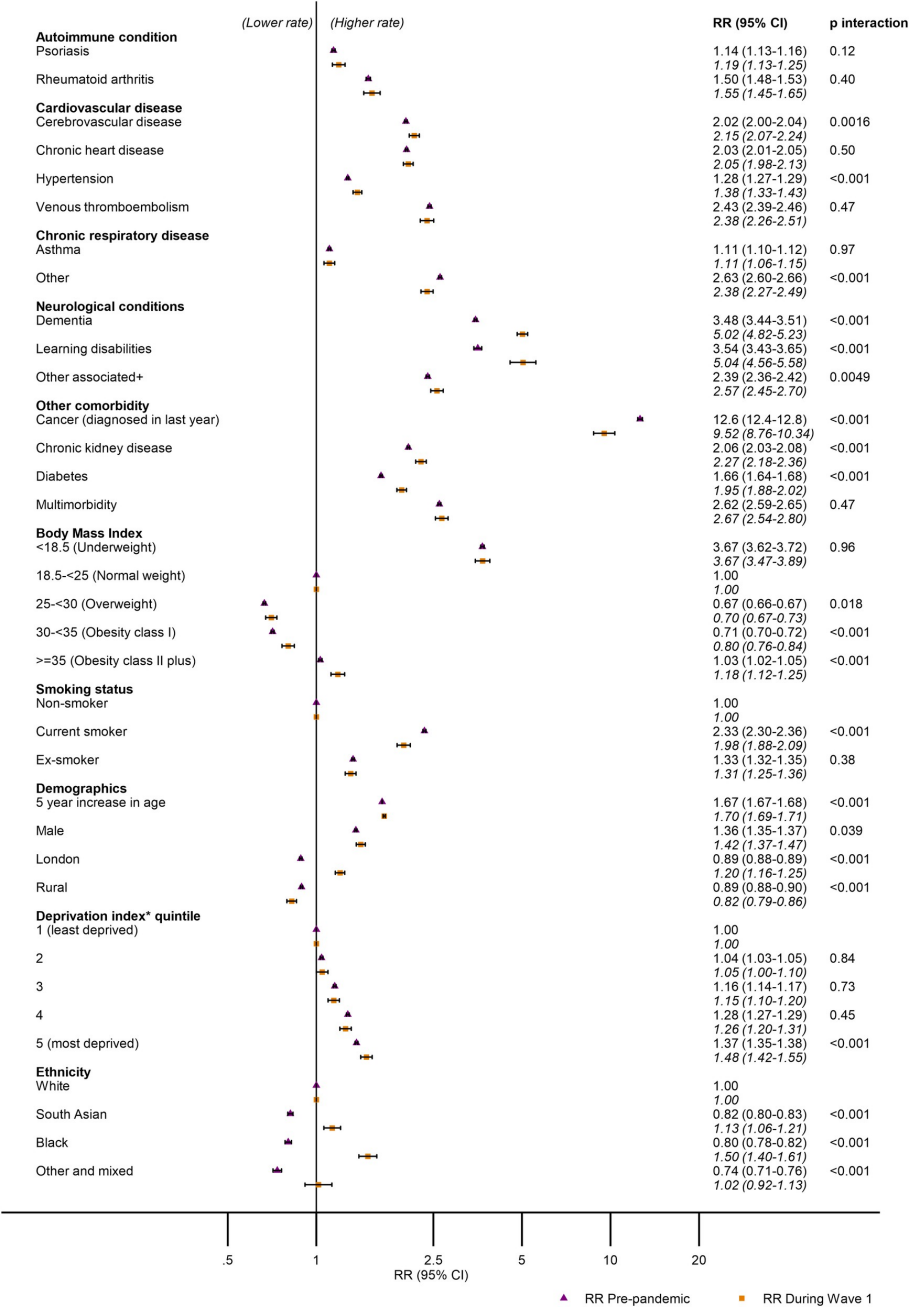
All cause RRs of death and 95% confidence intervals by morbidities, health, and demographic factors prepandemic and during Wave 1 adjusted for age, sex, season, and year. RR, relative rate. +with respiratory infections, *Carstairs Index (not available in Northern Ireland).

Fig 3 displays RRs of death for each factor before and during Wave 1, adjusted for age, sex, seasonality, and year. In most instances, there was strong evidence (*p* < 0.01) of small increases in the association between factors and mortality observed in the prepandemic period compared to Wave 1. For example, each 5-year increase in age was associated with 1.67 (95% CI 1.67 to 1.68) times greater rate of death in prepandemic and 1.70 (95% CI 1.69 to 1.71) times greater rate of death in Wave 1 of the pandemic (*p*-value for interaction between categorical age and the pandemic term < 0.01). RRs of death for each 5-year age category before and during Wave 1 are displayed in S3 Fig.

For a minority of factors, there were appreciable differences in the RR of death between the prepandemic and pandemic periods. Prepandemic, mortality rates were lower in London compared to other regions of the UK (RR 0.89, 95% CI 0.88 to 0.89), while in Wave 1, mortality rates were higher in London than in other regions of the UK (1.20, 95% CI 1.16 to 1.25). Similarly, compared to white ethnic groups, prepandemic mortality rates were lower in black (0.80, 95% CI 0.78 to 0.82), South Asian (0.81, 95% CI 0.80 to 0.83), and other non-white ethnic groups (0.66, 95% CI 0.64 to 0.69). However, during Wave 1 of the pandemic, RRs of death in minority ethnic groups were higher compared to white people (blacks 1.53, 95% CI 1.43 to 1.64; South Asians 1.15, 95% CI 1.08 to 1.23; other ethnicities 1.03, 95% 0.91 to 1.17).

One of the most striking findings was that there was a substantial increase in the RRs for dementia and learning difficulties in the Wave 1 compared to the prepandemic period. Prepandemic, people with dementia had a 3.47 (95% CI 3.44 to 3.51) times higher mortality rate than those without dementia, but this increased to 5.07 (95% CI 4.87 to 5.28) times higher in Wave 1. The equivalent estimates for learning difficulties were 3.55 (95% CI 3.44 to 3.51) prepandemic, increasing to 4.82 (95% CI 4.35 to 5.34) during Wave 1.

Cancer diagnosed in the last year was associated with elevated mortality rates both before the pandemic and during Wave 1, but this association was attenuated during Wave 1 (before pandemic: 11.1, 95% CI 10.9 to 11.3; during Wave 1: 8.37, 95% CI 7.74 to 9.05).

### Secondary and sensitivity analyses

S4 Fig shows RRs of death for alternative cancer definitions compared with those who never had cancer. RRs were slightly lower during Wave 1 than before the pandemic, except for haematological cancers diagnosed in the last year. S2 to S5 Tables show RRs by patient or database characteristics. We observed higher RRs before and during Wave 1 of the pandemic for people aged 40 to 69 compared to people age 70 or older for most risk factors (S2 Table). When comparing sexes (S3 Table) and databases (S4 Table), similar patterns were observed when comparing prepandemic RRs to Wave 1 RRs, except for ethnicity and region. Minimal differences were observed when using linked ONS mortality and HES APC data (S5 Table) except for cancer recently diagnosed, where higher RRs were observed when using the linked data prepandemic and multimorbidity where higher RRs were observed both prepandemic and during Wave 1.

S6 Table compares RRs in people of non-white ethnicity versus white ethnicity in London compared to the rest of the UK. The observed increased rates during Wave 1 were attenuated in both London and the rest of the UK compared to the primary analyses that included all UK practices.

S7 Table shows RRs for missing data categories. RRs for missing categories most closely resembled the baseline (larger) category (white ethnicity, nonsmokers, and normal weight), therefore indicating that their exclusion may not have led to major biases in the estimated RRs.

## Discussion

### Statement of principal findings

In our very large UK study population, the rate of death by any cause increased by 43% (95% CI 40% to 47%) during Wave 1 of the pandemic compared to that expected based on prepandemic levels and trends (2015 to 2019). There were small (+/− 10%) increases in the RR of death during Wave 1 associated with most of the factors we examined, compared to the prepandemic era. For example, chronic heart disease was associated with 2.03 (95% CI 2.01 to 2.05) times higher rate of death during Wave 1, while prepandemic, the RR was 2.05 (95% CI 1.98 to 2.13). However, bigger changes in the RR of death were seen for people with dementia or learning disabilities, with people in these groups having an approximate 5-fold increased rate of death during Wave 1, compared to people without these conditions, while prepandemic, this was 3.5 times higher. During Wave 1, we also observed an inversion of the mortality patterns by region and ethnicity: London registered the highest RR of death, while prepandemic had the lowest, and people of black or South Asian ethnicity had lower rates of death prepandemic, compared to the white population, but markedly increased rates during Wave 1.

### Strengths and weaknesses of the study

A major strength of this study is the use of 2 very large and well-characterised datasets of primary care electronic health records. This allowed us to estimate effects for a large and diverse range of chronic conditions, including cardiovascular, respiratory, neurological, and renal diseases, and rarer conditions that would be difficult to study otherwise. CPRD data are of good quality, with high completeness and validity reported for both diagnoses and recorded deaths [26,30]. The availability of data from several years prior to the pandemic permitted us to account for secular and seasonal trends in mortality, and to time-update exposures such as smoking status, obesity, and asthma. Finally, we carried out multiple sensitivity analyses to assess the robustness of the results.

However, this study also has limitations. There may have been misclassification of the vital status of a small number of individuals in some weeks due to imprecise recording of the exact date of death in CPRD. We expect this to have little impact as 98% of the death dates in CPRD are within 30 days of the ONS date of death [26] and our sensitivity analysis using the ONS death date yielded similar results. There is also a potential for misclassification of the exposures, as information may be incomplete (e.g., diagnoses from secondary care not coded in the primary care record) or inaccurate (e.g., patients in correctly reporting their smoking behaviour) and vary between practices. However, the similar results of the sensitivity analyses including hospital diagnoses in addition to primary care ones suggest minor impact for most conditions. Mortality rate ratios for cancer recently diagnosed and multimorbidity were, however, higher in linked data compared to when primary care data only were used, reflecting known under ascertainment of cancer in primary care data [31]. This was probably exacerbated by the reduced diagnostic activity during Wave 1 reported previously [32] and depicted in our graphs of person weeks contributing over the study period. Thus, our main results may underestimate the rates of death in people with cancer because of misclassification, especially during the pandemic. Even though CPRD data are representative of the UK population in terms of age and sex, they are not regionally representative and include few practices from Eastern England [22]. This, together with likely clustering of practices within regions by Clinical Commissioning Group (CCG), may explain differences observed in RRs for ethnicity and region in our sensitivity analyses separating the CPRD GOLD and CPRD Aurum databases. For analyses of health factors, we see no reasons to doubt that the pattern observed elsewhere in the UK would not be applicable. Our models for ethnicity, BMI, and smoking included only patients with information on these variables. Our findings from these complete cases analyses are nevertheless valid, under the assumption that missingness for these variables is conditionally independent of the outcome [33].

We should note that the excess mortality in Wave 1 reported in our study cannot be dissociated from the widespread efforts to suppress the virus transmission that involved a national lockdown and imposed social distancing. We cannot assume that the risks observed during Wave 1 would apply to other waves, as population behaviour, risk perception, and transmissibility of the virus most likely have changed.

### Strengths and weaknesses in relation to other studies

Our observation of an overall RR of death in Wave 1 compared to the prepandemic period of 1.43 (95% CI 1.40 to 1.47), following adjustment for seasonality, year, age, and sex, compares closely to a 47% increase in death observed in England and Wales during the same period in ONS mortality data compared to expected deaths based on an average from 2015 to 2019 (estimated from data provided by the ONS [34,35]).

There have been few studies of how excess mortality during the COVID-19 pandemic varied by health and wider demographic factors. de Lusignan and colleagues [17] reported excess mortality among patients from several demographic and clinical groups in England during Wave 1. Our results are broadly consistent with this previous study, but we are unable to directly compare the magnitude of the excess mortality due to methodological differences: In particular, the de Lusignan study used national life table data as an external basis for computing expected deaths in contrast to our use of prepandemic mortality within the same population. They were therefore unable to assess whether the relative risk of people with health and demographic factors differed in Wave 1 compared to previous years.

Our finding of an increased RR of death during Wave 1 of the pandemic in people with and without dementia and learning difficulties is consistent with cohort studies that have shown that excess mortality was higher in care home residents in England and Wales, especially those living in care homes catering for older people and those with dementia [12,36–38], and with ONS data that showed that 30% of all COVID-19–related deaths in England and Wales between March and June 2020 occurred in care homes, and 26% of all COVID-19–related deaths were in people with dementia [39]. Rates of SARS-CoV-2 infection were much higher among those living in care homes than among those living in private homes during Wave 1. This disproportionate exposure to SARS-CoV-2 may explain the increased mortality rate ratio in these 2 groups of the population and is consistent with studies conducted elsewhere that showed increased risk of infection among people with dementia [40]. However, we cannot rule out that factors other than infection may explain the increased risk of mortality. Joy and colleagues [41] quantified risk of mortality in a cohort of people with known SARS-CoV-2 infection status and found a similarly increased risk of mortality among people with learning disabilities and, compared to similar people without learning disabilities, suggesting that comorbidity and treatment may also explain part of the increased risk of mortality. We could not formally test these hypotheses, however, as we did not have data on the type of dwelling (collective versus private), and information on SARS-CoV-2 infection status in Wave 1 was limited by testing capacity.

Going in the other direction from dementia and learning disabilities, the RR of death among people with a recent diagnosis of cancer was lower during Wave 1 than it was prepandemic. This may be due to a lower risk of SARS-CoV-2 infection in cancer patients, compared to those without cancer consequent upon shielding. On 22 March 2020, the UK Government issued a list of preexisting clinical conditions that were considered at the time to put people at particularly high risk of serious disease or death if they contracted COVID-19, which included active cancer [42]. In July 2020, the ONS undertook a survey of clinically extremely vulnerable persons in England, concluding that 95% reported either completely or mostly following government shielding guidance [43].

Demographic factors associated with raised risk of exposure to SARS-CoV-2 may also explain the partially interrelated changes in the RR observed during Wave 1 for region and ethnicity. London is a global multicultural city with major international links and likely an important point of entry of imported SARS-CoV-2–infected cases. As the most densely populated region in the UK, with 5,701 people per squared kilometre [44], infection spread fast in London prior to the implementation of control measures [45]. The ethnic inequalities in COVID-19 mortality in Wave 1 in the UK have been reported consistently [7,8,17,41] and likely related to a complex interaction of factors including a predominance in some public facing occupations (e.g., food retail and healthcare), larger and often multigenerational households, and deprivation. Increases on excess mortality in ethnic minority groups have also been reported in the United States and Sweden [46–48]. Similar to other studies [16,41], our increase in the RR of mortality was disproportionately increased among those living in the most deprived areas, reflecting the perpetuation of the adverse effect of low socioeconomic status in health and mortality.

For many of the factors we examined, including most morbidities, there was little change in the RR of death in Wave 1 compared to prepandemic. This is in line with previous research suggesting similar strengths of associations with risk factors for mortality due to COVID-19 and other causes [49], but still striking. This firstly suggests that most of these characteristics are not particularly predictive of exposure to SARS-CoV-2. Beyond this, however, it can be interpreted as demonstrating that the net effect of COVID-19 in different subgroups of the population is to simply amplify baseline mortality risk by a constant amount. This is akin to David Spiegelhalter’s observation that the COVID-19 case-fatality age-curve in Wave 1 ran almost perfectly in parallel with the exponential increase with age in the risk of death prepandemic [50]. This has been interpreted as showing that COVID-19 has the effect of compressing one’s annual risk of death (whatever that may be) into fewer weeks. This insight, applied to our population study of excess deaths, suggests that the effect of the pandemic has been to accelerate the tempo of underlying mortality rate by a fixed proportional degree. However, the pathophysiological underpinning of this remains unclear and is beyond the scope of this study.

### Meaning of the study and future research

This study has implications for clinical practice, policy, and future research. For most morbidities, the RR did not change very much because of the pandemic, which means that from a clinical perspective, prepandemic knowledge about the relative frailty associated with different conditions can be reasonably applied in the pandemic situation. However, the high mortality observed in some vulnerable groups, such as those with dementia and learning disabilities (many of whom live in institutions), should be a learning moment for the COVID-19 or other pandemic, and preventive measures should be implemented to avoid the spread of potentially fatal infectious agents. The RRs of death for characteristics that are strongly affected by population behaviour and the regional epidemiology of the virus may change as the pandemic progresses. Future research may clarify whether there were differences across waves in the UK, particularly for factors such as ethnicity and deprivation, and investigate independent effects of individual health and demographic risk factors (e.g., ethnicity and health status).

## Supporting information

S1 Text Approved protocol and changes to approved protocol. (PDF)

S2 Text The Record Statement. Checklist of items, extended from the STROBE statement, which should be reported in observational studies using routinely collected health data.
(PDF)

S3 Text Risk factor definitions. (PDF)

S4 Text Estimation equation for the basic model. (PDF)

S1 Fig Millions of person-weeks contributed over the study period for the study population and specified medical conditions. Red dotted lines: start and end of Wave 1.
(TIF)

S2 Fig Observed and predicted deaths per million person-weeks for full study period for study period. Green dots: observed deaths per million person-weeks; solid grey line: predicted deaths per million person-weeks from the basic model restricted to prepandemic period; dotted grey lines: start and end of Wave 1 (5 March to 27 May 2020). Y-axis scale differs for each graph.
(TIF)

S3 Fig All-cause RRs of death and 95% CIs by 5-year age category prepandemic and during Wave 1 adjusted for age, sex, season, and year. CI, confidence interval; RR, rate ratio.
(TIF)

S4 Fig All-cause RRs of death and 95% CIs in people with cancer compared to people without cancer prepandemic and during Wave 1 adjusted for age, sex, season, and year. CI, confidence interval; RR, rate ratio.
(TIF)

S1 Table Distribution of deviance residuals in the prepandemic period for the basic model. (PDF)

S2 Table All-cause RRs of death and 95% CIs by morbidities, health, and demographic factors prepandemic and during Wave 1 adjusted for age, sex, season, and year by age group. CI, confidence interval; RR, rate ratio.
(PDF)

S3 Table All-cause RRs of death and 95% CIs by morbidities, health, and demographic factors prepandemic and during Wave 1 adjusted for age, sex, season, and year by sex. CI, confidence interval; RR, rate ratio.
(PDF)

S4 Table All-cause RRs of death and 95% CIs by morbidities, health, and demographic factors prepandemic and during Wave 1 adjusted for age, sex, season, and year by database. CI, confidence interval; RR, rate ratio.
(PDF)

S5 Table All-cause RRs of death and 95% CIs by morbidities, health, and demographic factors prepandemic and during Wave 1 adjusted for age, sex, season, and year with and without linked data. CI, confidence interval; RR, rate ratio.
(PDF)

S6 Table All-cause RRs of death and 95% CIs by ethnicity in the full study population, London, and the rest of the UK prepandemic and during Wave 1 adjusted for age, sex, season, and year by age group. CI, confidence interval; RR, rate ratio.
(PDF)

S7 Table All-cause RRs of death and 95% CIs by ethnicity, BMI, and smoking status groups with a missing category prepandemic and during Wave 1 adjusted for age, sex, season, and year by age group. BMI, body mass index; CI, confidence interval; RR, rate ratio.
(PDF)

## Abbreviations

BMI: body mass index
CCG: Clinical Commissioning Group
CI: confidence interval
COVID-19: Coronavirus Disease 2019
CPRD: Clinical Practice Research Datalink
GP: general practitioner
HES APC: Hospital Episode Statistics Admitted Patient Care
ONS: Office for National Statistics
RR: relative rate
SARS-CoV-2: Severe Acute Respiratory Syndrome Coronavirus 2
SD: Standard Deviation
UK: United Kingdom
