# Supplementary material for: Factors associated with excess all-cause mortality in the first wave of the COVID-19 pandemic in the UK: A time series analysis using the Clinical Practice Research Datalink
Source: PLoS Med. 2022 Jan 6;19(1):e1003870. doi: 10.1371/journal.pmed.1003870 (PMC8735664; doi:10.1371/journal.pmed.1003870)
Supplement: S3 Text — (PDF) [file pmed.1003870.s003.pdf]

# Risk factor definitions

| Risk factor | Definition | Derivation |
|---|---|---|
| **Demographic** | | |
| Age | 5-year age groups from 40-<45 to ≥ 90 | 01/07/birthyear (Exact date of birth not collected by CPRD to maintain the de-identified nature of the data) |
| Sex | Male, Female | Recorded in primary care record |
| Region | Value to indicate where in the UK the practice is based. The region denotes the former Strategic Health Authority for practices within England, and the country i.e. Wales, Scotland, or Northern Ireland for the rest | Provided by CPRD |
| Practice area-based deprivation | Carstairs Index quintile used as proxy for socio-economic status (1 least deprived, 2, 3, 4, 5 most deprived) | Carstairs Index quintile identified by CPRD through linkage to practice postcode |
| Patient area-based deprivation | Replaces practice area-based deprivation in sensitivity analysis using linked data | Carstairs Index quintile identified by CPRD through third party linkage to patient postcode |
| Rural-Urban | Rural or Urban | Identified by CPRD through linkage of Rural Urban Classifications for England & Wales, Scotland and Northern Ireland to practice postcode. Mixed Urban-Rural in Northern Ireland reclassified as Urban. |
| Ethnicity | 5 categories, as defined in the Census: White; Mixed or multiple ethnic groups; Asian or Asian British; Black, African, Caribbean or Black British; Other. | Derived from information in the patient clinical records in analyses of primary care data. In analyses of primary care data linked to hospital data, ethnicity variable was further supplemented with the information in the hospital record. (https://academic.oup.com/jpubhealth/article/36/4/684/1529704) |
| **Morbidity** | | |
| **Cardiovascular** | | |
| Chronic heart disease | Includes ischaemic heart disease, congenital heart disease, heart failure/cardiomyopathy, valve diseases, and primary hypertension | Any coded previous clinical diagnosis, major intervention for, or clinical review for chronic heart disease. Codes from primary and secondary care included in the sensitivity analysis. |
| Cerebrovascular disease | Stroke or transient ischaemic attack | Any coded previous clinical diagnosis, major intervention for, or clinical review for cerebrovascular disease. Codes from primary and secondary care included in the sensitivity analysis. |
| Venous thrombo-embolism | Venous thrombo-embolism | Any coded previous clinical diagnosis, major intervention for, or clinical review for venous thrombo-embolism. Codes from primary and secondary care included in the sensitivity analysis. |
| Hypertension | Hypertension | Diagnosis code or two abnormal blood pressure records (systolic blood pressure ≥140 mmHg or diastolic blood pressure ≥90 mmHg) within a 2-year period. Assumed to be chronic. (https://www.ncbi.nlm.nih.gov/pmc/articles/PMC5072168/) |
| **Respiratory** | | |
| Asthma | Current asthma diagnosis | Specific record of asthma diagnosis in last 3 years. (Validation of asthma recording in the Clinical Practice Research Datalink (CPRD) \| BMJ Open). Codes from primary and secondary care included in the sensitivity analysis. |

| | | |
|---|---|---|
| Other chronic respiratory condition | Chronic obstructive pulmonary disease (COPD), emphysema, bronchitis, cystic fibrosis, or fibrosing interstitial lung diseases. | Any previous coded diagnosis or clinical review. Codes from primary and secondary care included in the sensitivity analysis. |
| **Autoimmune** | | |
| Rheumatoid Arthritis | Rheumatoid arthritis | Any previous coded diagnosis or clinical review |
| Lupus erythematosus | Lupus erythematosus | Any previous coded diagnosis or clinical review. Excluded from analysis due to problems with model convergence. |
| Psoriasis | Psoriasis | Any previous coded diagnosis or clinical review |
| **Neurological** | | |
| Learning / intellectual disability | Learning / intellectual disability | Any previous coded diagnosis or clinical review. Codes from primary and secondary care included in the sensitivity analysis. |
| Dementia | Dementia | Any previous coded diagnosis or clinical review. Codes from primary and secondary care included in the sensitivity analysis. |
| Other chronic neurological condition associated with respiratory infection | Parkinson's disease, multiple sclerosis, motor neurone disease, cerebral palsy, quadriplegia, progressive cerebellar disease, Huntington's, poliomyelitis, and myasthenia | Any previous coded diagnosis or clinical review. Codes from primary and secondary care included in the sensitivity analysis. |
| **Other** | | |
| Chronic liver disease | Cirrhosis, oesophageal varices, biliary atresia and chronic hepatitis. | Any previous coded diagnosis of or clinical review. Excluded from analysis due to problems with model convergence. |
| Chronic Kidney Disease (CKD) | CKD, history of dialysis or renal transplant. Or with estimated glomerular filtration rate (eGFR) to classify CKD stage. | Any previous coded diagnosis of or clinical review of CKD, dialysis, or renal transplant. Any previous serum creatinine record that allows the calculation of an estimated glomerular filtration rate (eGFR) to classify CKD stage 3a-5.<br><br>Codes from primary and secondary care included in the sensitivity analysis. |
| Diabetes | Diabetes mellitus excluding gestational diabetes | Any previous coded diagnosis of or clinical review. Codes from primary and secondary care included in the sensitivity analysis. |
| Recently diagnosed cancer | All first malignant tumours diagnosed within the last year | First ever malignant cancer diagnosis diagnosed in the last year (time-updating). Not counted if there is a previous record of a historical or secondary malignancy. Codes from primary and secondary care included in the sensitivity analysis. |
| Permanent immunosuppression | HIV, solid organ transplant or other permanent immunosuppression (such as genetic conditions compromising immune function). | Any previous coded diagnosis of or clinical review. Planned to include in Hospital Episode Statistics (HES) sensitivity analysis. Excluded from analysis due to problems with model convergence. |
| **Multimorbidity** | | |
| Multimorbidity | More than one condition of the following: asthma, other chronic respiratory disease, chronic heart disease, chronic kidney disease, chronic liver disease, chronic neurological disease, diabetes or permanent immunosuppression | Counted from diagnosis of second condition within list. |
| **Health Indicators** | | |
| Smoking Status | Non-smoker, smoker, ex-smoker | Previous structured or coded record of smoking status (time updating). Non-smokers recategorised as ex-smoker if there was a previous record of smoking |

| | | |
|---|---|---|
| Body Mass Index (BMI) | Categorised based on WHO categories for Caucasian populations | Derived from weight and height measures (BMI category time updating). (https://www.ncbi.nlm.nih.gov/pubmed/24038008) |
| **Cancer (secondary analyses)** | | |
| Haematological Cancer | Lymphoma, multiple myeloma and leukaemia, and other haematological cancers | First ever coded record for malignant cancer identifies haematological malignancy. Not counted if there is a previous record of a historical or secondary malignancy. Separate variables for:<br>- Above record recorded in last year (time-updating)<br>- Above record recorded 1 to 5 years ago (time-updating)<br>- Above record recorded more than 5 years ago |
| Non-haematological Cancer | All other malignant cancers | First ever coded record for malignant cancer identifies non-haematological malignancy. Not counted if there is a previous record of a historical or secondary malignancy. Separate variables for:<br>- Above record recorded in last year (time-updating)<br>- Above record recorded 1 to 5 years ago (time-updating)<br>- Above record recorded more than 5 years ago |
