# Supplementary material for: Factors associated with excess all-cause mortality in the first wave of the COVID-19 pandemic in the UK: A time series analysis using the Clinical Practice Research Datalink
Source: PLoS Med. 2022 Jan 6;19(1):e1003870. doi: 10.1371/journal.pmed.1003870 (PMC8735664; doi:10.1371/journal.pmed.1003870)
Supplement: S4 Text — (PDF) [file pmed.1003870.s004.pdf]

Estimation equation for the basic model

$\log(\mu) = \beta_0 + \beta_1(\text{year} - 2017) + \beta_2([\text{year}-2017]^\wedge 2) + \beta_3(\sin[\text{pi x degree}/180]) + \beta_4 (\sin[2 \text{ x pi x degree}/180]) + \beta_5(\sin[3 \text{ x pi x degree}/180]) + \beta_6(\cos[\text{pi x degree}/180]) + \beta_7 (\cos[2 \text{ x pi x degree}/180]) + \beta_8 (\cos[3 \text{ x pi x degree}/180])$

$\text{Var}(\mu)= \mu + \alpha \mu^2$

Where the unit of observation is in weeks:

$\mu=E(y/N)$

y = deaths during the unit of observation (count)

N= number of patients during the unit of observation

degree = (week/52)*360
