# Supplementary figures and images for: Factors associated with excess all-cause mortality in the first wave of the COVID-19 pandemic in the UK: A time series analysis using the Clinical Practice Research Datalink
Source: PLoS Med. 2022 Jan 6;19(1):e1003870. doi: 10.1371/journal.pmed.1003870 (PMC8735664; doi:10.1371/journal.pmed.1003870)

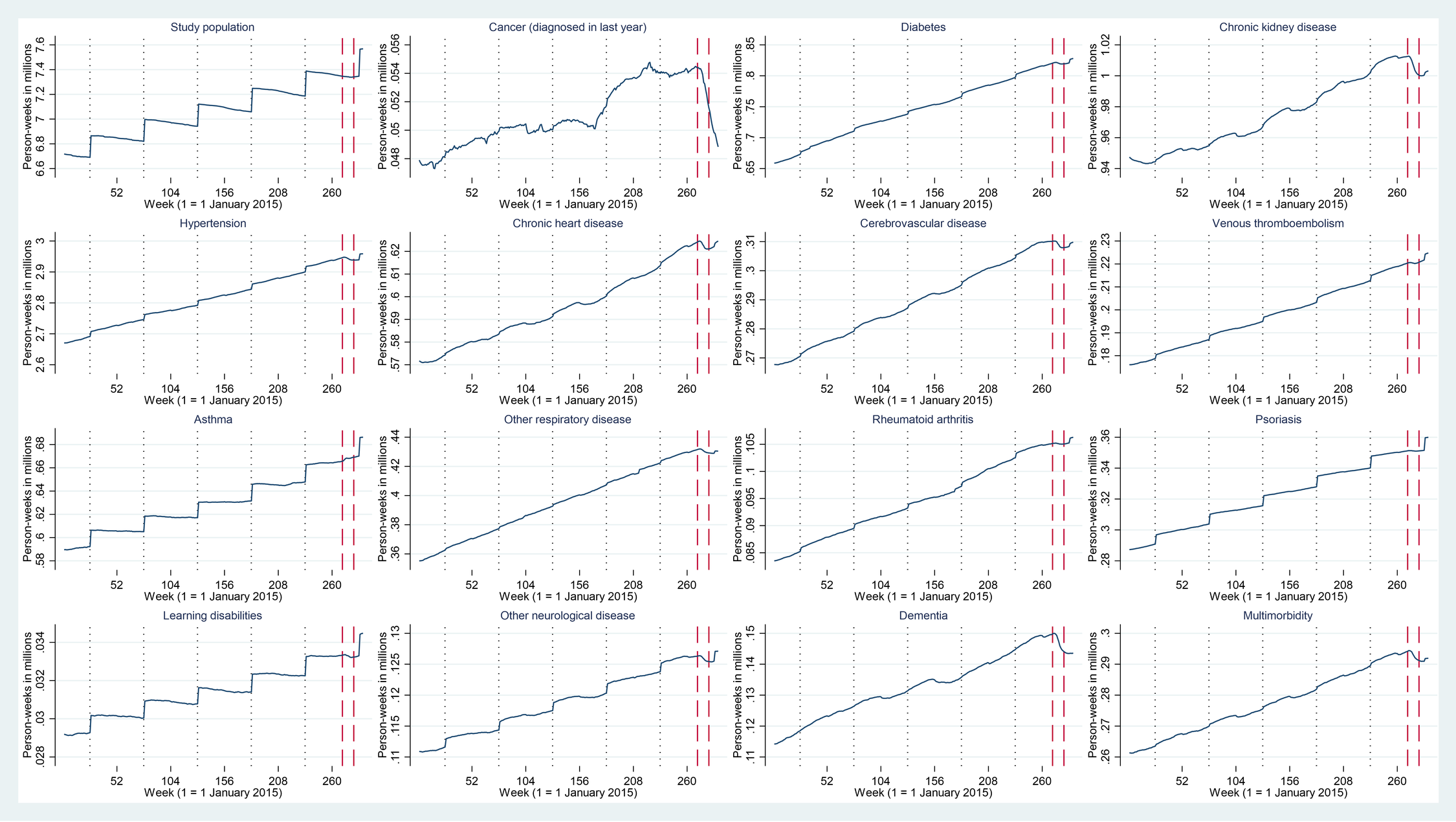

Supplement: S1 Fig — Red dotted lines: start and end of Wave 1. (TIF) [file pmed.1003870.s005.tif]

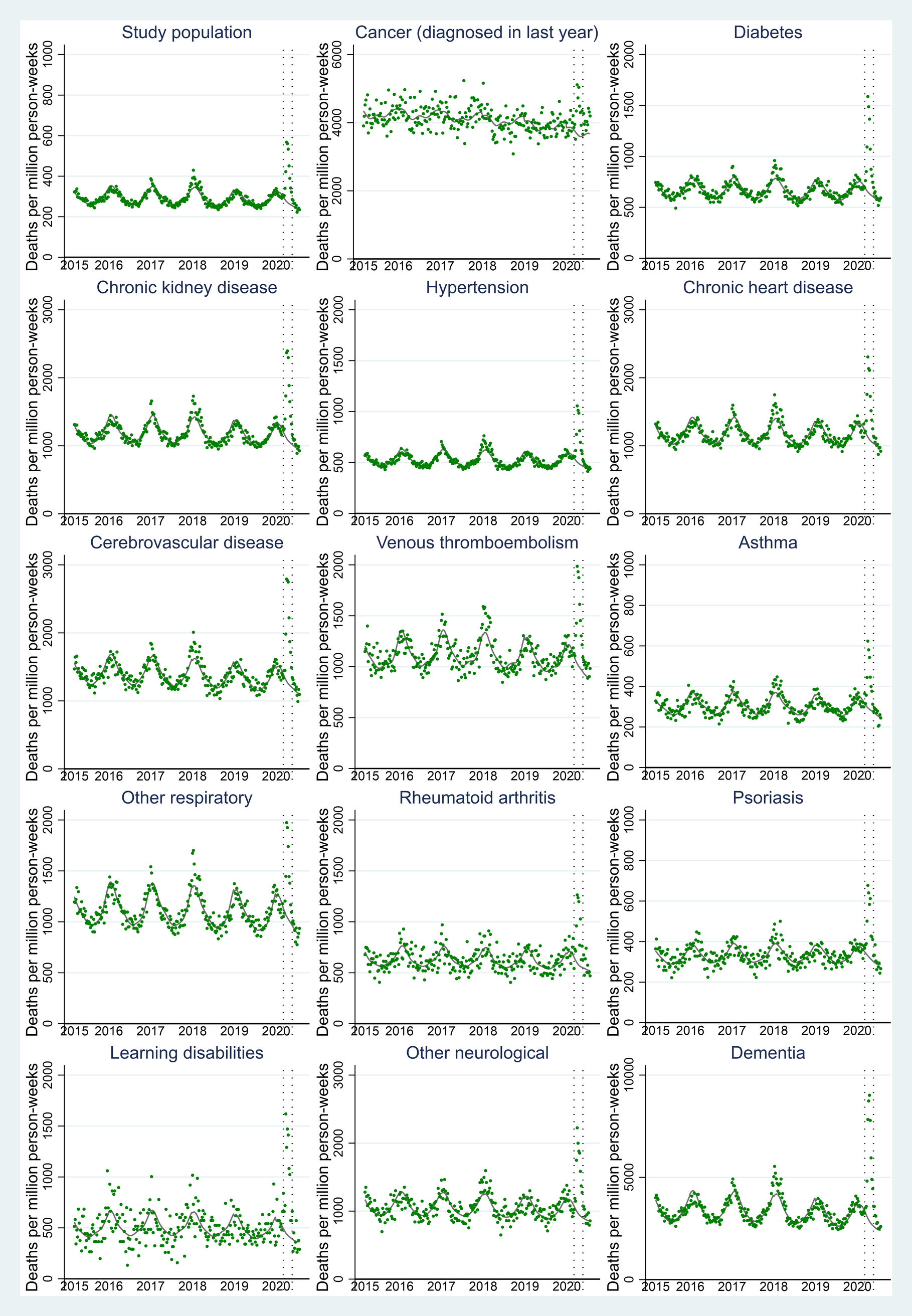

Supplement: S2 Fig — Green dots: observed deaths per million person-weeks; solid grey line: predicted deaths per million person-weeks from the basic model restricted to prepandemic period; dotted grey lines: start and end of Wave 1 (5 March to 27 May 2020). Y-axis scale differs for each graph. (TIF) [file pmed.1003870.s006.tif]

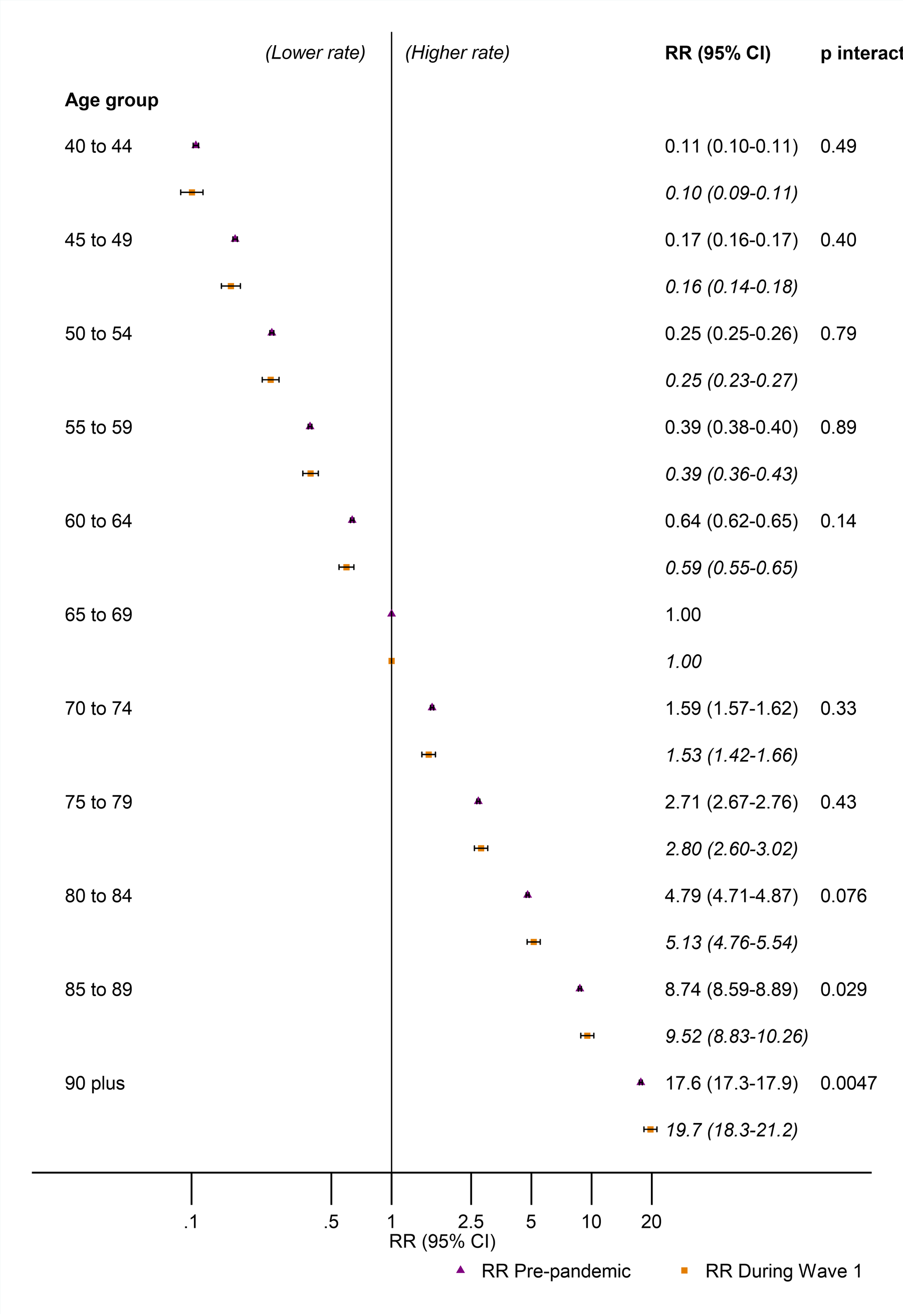

Supplement: S3 Fig — CI, confidence interval; RR, rate ratio. (TIF) [file pmed.1003870.s007.tif]

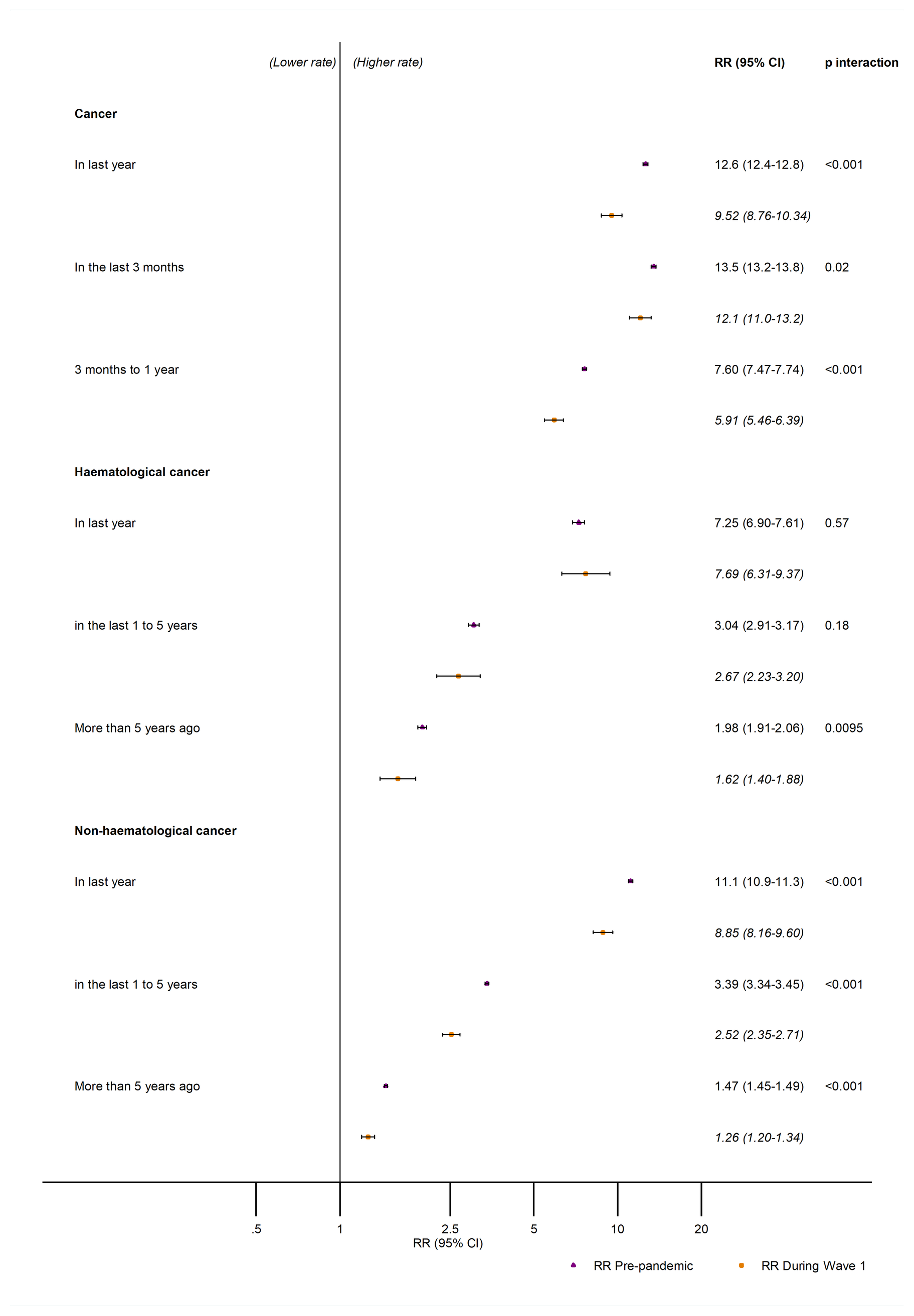

Supplement: S4 Fig — CI, confidence interval; RR, rate ratio. (TIF) [file pmed.1003870.s008.tif]
