# Supplementary material for: Factors associated with excess all-cause mortality in the first wave of the COVID-19 pandemic in the UK: A time series analysis using the Clinical Practice Research Datalink
Source: PLoS Med. 2022 Jan 6;19(1):e1003870. doi: 10.1371/journal.pmed.1003870 (PMC8735664; doi:10.1371/journal.pmed.1003870)
Supplement: S1 Table — (PDF) [file pmed.1003870.s009.pdf]

S1 Table: Distribution of deviance residuals in the pre-pandemic period for the basic model

Factor	Mean weekly deaths (sd)	Mean weekly absolute deviance (sd)	Mean (sd) weekly absolute deviance as a proportion of weekly deaths
Study population	2,042.4 (235.9)	86.1 (77.7)	0.04 (0.03)
Hypertension	1,461.9 (177.1)	64.9 (58.2)	0.04 (0.03)
Chronic kidney disease	1,143.1 (144.8)	53.1 (47.5)	0.05 (0.04)
Chronic heart disease	694.5 (88.5)	35.8 (29.4)	0.05 (0.04)
Diabetes	493.2 (65.1)	26.9 (21.5)	0.05 (0.04)
Dementia	441.0 (77.4)	30.1 (26.5)	0.07 (0.05)
Other respiratory	435.7 (64.1)	24.5 (20.3)	0.06 (0.04)
Multimorbidity	399.1 (54.9)	22.1 (17.4)	0.06 (0.04)
Cerebrovascular disease	397.4 (50.5)	22.7 (17.8)	0.06 (0.04)
Venous thromboembolism	218.5 (30.5)	14.9 (11.0)	0.07 (0.05)
Cancer (diagnosed in last year)	209.9 (16.8)	12.3 (9.9)	0.06 (0.05)
Asthma	190.1 (28.8)	13.8 (10.9)	0.07 (0.06)
Other neurological	124.6 (19.2)	10.7 (8.1)	0.09 (0.07)
Psoriasis	106.1 (16.1)	9.1 (6.6)	0.09 (0.06)
Rheumatoid arthritis	58.8 (10.1)	6.3 (4.8)	0.11 (0.09)
Learning disabilities	15.8 (4.9)	3.4 (2.6)	0.24 (0.27)
