# Supplementary material for: Factors associated with excess all-cause mortality in the first wave of the COVID-19 pandemic in the UK: A time series analysis using the Clinical Practice Research Datalink
Source: PLoS Med. 2022 Jan 6;19(1):e1003870. doi: 10.1371/journal.pmed.1003870 (PMC8735664; doi:10.1371/journal.pmed.1003870)
Supplement: S2 Table — CI, confidence interval; RR, rate ratio. (PDF) [file pmed.1003870.s010.pdf]

S2 Table: All-cause relative rates of death and 95% confidence intervals by morbidities, health and demographic factors pre-pandemic and during Wave 1 adjusted for age, sex, season and year by age group

	Pre-pandemic		During Wave 1	
	**40 to 69**	**70 or older**	**40 to 69**	**70 or older**
**DEMOGRAPHICS**				
**Age**				
5-year increase in age	1.57 (1.57-1.58)	1.82 (1.81-1.83)	1.58 (1.55-1.61)	1.88 (1.85-1.92)
**Sex**				
Female	1.00	1.00	1.00	1.00
Male	1.45 (1.43-1.47)	1.32 (1.31-1.33)	1.56 (1.48-1.64)	1.36 (1.30-1.42)
**Carstairs deprivation index quintile**				
1 (least deprived)	1.00	1.00	1.00	1.00
2	1.07 (1.04-1.09)	1.04 (1.02-1.05)	1.12 (1.02-1.23)	1.03 (0.98-1.09)
3	1.26 (1.23-1.29)	1.13 (1.11-1.14)	1.22 (1.11-1.33)	1.13 (1.08-1.19)
4	1.50 (1.46-1.53)	1.21 (1.20-1.23)	1.46 (1.33-1.59)	1.21 (1.15-1.27)
5 (most deprived)	1.63 (1.60-1.67)	1.28 (1.27-1.30)	1.80 (1.65-1.96)	1.39 (1.32-1.46)
**Ethnicity**				
Black	0.82 (0.79-0.86)	0.79 (0.77-0.82)	1.59 (1.42-1.78)	1.45 (1.33-1.58)
Other and mixed	0.65 (0.61-0.68)	0.80 (0.77-0.83)	0.93 (0.78-1.11)	1.08 (0.95-1.24)
South Asian	0.71 (0.68-0.73)	0.88 (0.86-0.90)	1.10 (0.99-1.23)	1.15 (1.06-1.25)
White	1.00	1.00	1.00	1.00
**Region**				
London	0.85 (0.84-0.87)	0.90 (0.89-0.91)	1.18 (1.11-1.26)	1.21 (1.15-1.26)
Other	1.00	1.00	1.00	1.00
**Urban Rural**				
Rural	0.79 (0.78-0.81)	0.92 (0.92-0.93)	0.71 (0.66-0.77)	0.85 (0.81-0.89)
Urban	1.00	1.00	1.00	1.00
**HEALTH BEHAVIOURS / INDICATORS**				
**Body Mass Index**				
<18.5 (Underweight)	5.89 (5.74-6.04)	3.19 (3.14-3.24)	5.59 (5.01-6.23)	3.15 (2.96-3.35)
18.5-<25 (Normal weight)	1.00	1.00	1.00	1.00
25-<30 (Overweight)	0.65 (0.64-0.66)	0.67 (0.66-0.68)	0.76 (0.71-0.82)	0.67 (0.64-0.71)
30-<35 (Obesity class I)	0.72 (0.70-0.73)	0.70 (0.69-0.71)	0.93 (0.86-1.01)	0.74 (0.70-0.79)
>=35 (Obesity class II plus)	1.15 (1.13-1.18)	0.95 (0.94-0.97)	1.64 (1.52-1.77)	0.96 (0.89-1.02)
**Smoking status**				
Current smoker	3.40 (3.34-3.46)	1.85 (1.82-1.88)	2.90 (2.69-3.12)	1.56 (1.46-1.66)
Ex-smoker	1.67 (1.65-1.70)	1.21 (1.20-1.22)	1.63 (1.53-1.74)	1.19 (1.13-1.25)
Non-smoker	1.00	1.00	1.00	1.00
**MORBIDITY**				
**Autoimmune condition**				
Lupus erythematosus	2.19 (2.01-2.38)	1.47 (1.39-1.56)	1.33 (0.87-2.05)	1.17 (0.92-1.48)
Psoriasis	1.26 (1.23-1.30)	1.10 (1.09-1.12)	1.28 (1.16-1.41)	1.15 (1.08-1.22)
Rheumatoid arthritis	1.70 (1.63-1.77)	1.46 (1.44-1.49)	2.01 (1.74-2.32)	1.43 (1.33-1.54)
**Cardiovascular disease**				
Cerebrovascular disease	3.17 (3.10-3.24)	1.84 (1.82-1.86)	3.24 (2.99-3.52)	1.91 (1.83-2.00)
Chronic heart disease	2.98 (2.93-3.03)	1.80 (1.78-1.81)	3.06 (2.86-3.27)	1.75 (1.68-1.82)
Hypertension	1.57 (1.55-1.60)	1.14 (1.13-1.15)	1.82 (1.73-1.92)	1.16 (1.11-1.21)
Venous thromboembolism	4.99 (4.89-5.09)	1.80 (1.78-1.83)	4.64 (4.29-5.01)	1.74 (1.65-1.84)
**Chronic respiratory disease**				
Asthma	1.28 (1.25-1.30)	1.05 (1.04-1.06)	1.28 (1.19-1.38)	1.03 (0.98-1.09)
Other	4.15 (4.08-4.22)	2.16 (2.13-2.18)	3.72 (3.49-3.98)	1.89 (1.79-1.99)
**Neurological conditions**				
Dementia	6.85 (6.64-7.06)	3.25 (3.22-3.29)	7.77 (7.01-8.62)	4.73 (4.51-4.96)
Learning disabilities	4.54 (4.36-4.72)	2.60 (2.48-2.74)	6.46 (5.66-7.37)	3.90 (3.33-4.56)
Other associated with respiratory infections	3.69 (3.60-3.78)	2.15 (2.12-2.18)	3.84 (3.48-4.25)	2.29 (2.17-2.42)
**Other comorbidity**				
Cancer (diagnosed in last year)	26.4 (26.0-26.9)	6.85 (6.73-6.97)	20.3 (18.7-21.9)	5.01 (4.63-5.43)
Chronic kidney disease	3.42 (3.36-3.47)	1.70 (1.68-1.71)	3.97 (3.72-4.23)	1.76 (1.69-1.83)
Diabetes	2.27 (2.23-2.30)	1.46 (1.45-1.48)	2.89 (2.73-3.07)	1.61 (1.54-1.68)
Multimorbidity	4.77 (4.68-4.86)	2.12 (2.09-2.14)	5.18 (4.82-5.57)	1.99 (1.89-2.10)
