# Supplementary material for: Factors associated with excess all-cause mortality in the first wave of the COVID-19 pandemic in the UK: A time series analysis using the Clinical Practice Research Datalink
Source: PLoS Med. 2022 Jan 6;19(1):e1003870. doi: 10.1371/journal.pmed.1003870 (PMC8735664; doi:10.1371/journal.pmed.1003870)
Supplement: S3 Table — CI, confidence interval; RR, rate ratio. (PDF) [file pmed.1003870.s011.pdf]

S3 Table: All-cause relative rates of death and 95% confidence intervals by morbidities, health and demographic factors pre-pandemic and during Wave 1 adjusted for age, sex, season and year by sex

	Pre-pandemic		During Wave 1	
	**Male**	**Female**	**Male**	**Female**
**DEMOGRAPHICS**				
**Age**				
5-year increase in age	1.65 (1.65-1.66)	1.70 (1.70-1.70)	1.68 (1.66-1.69)	1.73 (1.71-1.75)
**Carstairs deprivation index quintile**				
1 (least deprived)	1.00	1.00	1.00	1.00
2	1.05 (1.03-1.06)	1.04 (1.02-1.05)	1.08 (1.01-1.15)	1.02 (0.95-1.08)
3	1.16 (1.14-1.18)	1.15 (1.13-1.17)	1.18 (1.11-1.25)	1.11 (1.05-1.19)
4	1.30 (1.28-1.32)	1.26 (1.24-1.28)	1.31 (1.23-1.39)	1.20 (1.13-1.28)
5 (most deprived)	1.39 (1.37-1.41)	1.34 (1.32-1.36)	1.54 (1.45-1.64)	1.41 (1.33-1.50)
**Ethnicity**				
Black	0.80 (0.78-0.83)	0.80 (0.78-0.83)	1.70 (1.56-1.86)	1.26 (1.13-1.41)
Other and mixed	0.72 (0.69-0.76)	0.75 (0.72-0.79)	1.08 (0.94-1.24)	0.94 (0.80-1.11)
South Asian	0.80 (0.78-0.82)	0.84 (0.82-0.87)	1.17 (1.08-1.28)	1.07 (0.97-1.19)
White	1.00	1.00	1.00	1.00
**Region**				
London	0.90 (0.89-0.91)	0.87 (0.86-0.88)	1.26 (1.20-1.32)	1.14 (1.08-1.20)
Other	1.00	1.00	1.00	1.00
**Urban Rural**				
Rural	0.88 (0.87-0.89)	0.90 (0.89-0.91)	0.82 (0.78-0.86)	0.83 (0.79-0.88)
Urban	1.00	1.00	1.00	1.00
**HEALTH BEHAVIOURS / INDICATORS**				
**Body Mass Index**				
<18.5 (Underweight)	4.26 (4.18-4.34)	3.31 (3.25-3.37)	3.99 (3.69-4.32)	3.48 (3.24-3.73)
18.5-<25 (Normal weight)	1.00	1.00	1.00	1.00
25-<30 (Overweight)	0.61 (0.60-0.62)	0.73 (0.72-0.74)	0.64 (0.61-0.68)	0.76 (0.71-0.81)
30-<35 (Obesity class I)	0.65 (0.64-0.66)	0.77 (0.76-0.79)	0.72 (0.68-0.77)	0.87 (0.82-0.93)
>=35 (Obesity class II plus)	0.95 (0.93-0.96)	1.11 (1.09-1.13)	1.05 (0.98-1.13)	1.29 (1.20-1.39)
**Smoking status**				
Current smoker	2.52 (2.48-2.56)	2.14 (2.11-2.18)	2.01 (1.88-2.15)	1.91 (1.78-2.06)
Ex-smoker	1.42 (1.40-1.44)	1.26 (1.24-1.28)	1.35 (1.28-1.43)	1.26 (1.19-1.33)
Non-smoker	1.00	1.00	1.00	1.00
**MORBIDITY**				
**Autoimmune condition**				
Lupus erythematosus	1.67 (1.52-1.83)	1.63 (1.54-1.72)	1.50 (1.03-2.19)	1.14 (0.89-1.46)
Psoriasis	1.14 (1.12-1.16)	1.14 (1.12-1.16)	1.17 (1.10-1.26)	1.20 (1.11-1.28)
Rheumatoid arthritis	1.46 (1.42-1.50)	1.51 (1.48-1.55)	1.55 (1.40-1.72)	1.54 (1.42-1.67)
**Cardiovascular disease**				
Cerebrovascular disease	1.94 (1.92-1.97)	2.06 (2.03-2.09)	2.04 (1.94-2.15)	2.23 (2.11-2.36)
Chronic heart disease	1.91 (1.89-1.93)	2.14 (2.11-2.17)	1.89 (1.80-1.97)	2.24 (2.11-2.38)
Hypertension	1.30 (1.28-1.31)	1.25 (1.23-1.26)	1.41 (1.35-1.47)	1.34 (1.27-1.41)
Venous thromboembolism	2.28 (2.24-2.31)	2.54 (2.50-2.59)	2.19 (2.05-2.34)	2.56 (2.36-2.77)
**Chronic respiratory disease**				
Asthma	1.09 (1.07-1.10)	1.12 (1.10-1.13)	1.06 (1.00-1.13)	1.14 (1.08-1.21)
Other	2.54 (2.50-2.57)	2.72 (2.67-2.76)	2.25 (2.12-2.39)	2.50 (2.33-2.68)
**Neurological conditions**				
Dementia	3.31 (3.27-3.36)	3.62 (3.56-3.68)	4.75 (4.50-5.01)	5.31 (4.99-5.64)
Learning disabilities	3.32 (3.18-3.46)	3.83 (3.66-4.01)	4.58 (4.00-5.24)	5.64 (4.85-6.56)
Other associated with respiratory infections	2.41 (2.37-2.45)	2.34 (2.30-2.39)	2.60 (2.44-2.77)	2.49 (2.31-2.67)
**Other comorbidity**				
Cancer (diagnosed in last year)	11.8 (11.4-12.1)	13.4 (13.0-13.7)	8.66 (7.69-9.76)	10.4 (9.3-11.6)
Chronic kidney disease	2.11 (2.09-2.14)	1.97 (1.94-2.00)	2.29 (2.17-2.41)	2.20 (2.08-2.34)
Diabetes	1.61 (1.59-1.63)	1.70 (1.68-1.73)	1.89 (1.80-1.98)	1.98 (1.87-2.10)
Multimorbidity	2.55 (2.51-2.59)	2.66 (2.61-2.71)	2.50 (2.35-2.66)	2.81 (2.61-3.03)
