# Supplementary material for: Factors associated with excess all-cause mortality in the first wave of the COVID-19 pandemic in the UK: A time series analysis using the Clinical Practice Research Datalink
Source: PLoS Med. 2022 Jan 6;19(1):e1003870. doi: 10.1371/journal.pmed.1003870 (PMC8735664; doi:10.1371/journal.pmed.1003870)
Supplement: S4 Table — CI, confidence interval; RR, rate ratio. (PDF) [file pmed.1003870.s012.pdf]

S4 Table: All-cause relative rates of death and 95% confidence intervals by morbidities, health and demographic factors pre-pandemic and during Wave 1 adjusted for age, sex, season and year by database

	Pre-pandemic		During Wave 1	
	CPRD GOLD	CPRD Aurum	CPRD GOLD	CPRD Aurum
**DEMOGRAPHICS**				
**Age**				
5-year increase in age	1.66 (1.66-1.67)	1.68 (1.68-1.69)	1.67 (1.65-1.69)	1.72 (1.70-1.73)
**Sex**				
Female	1.00	1.00	1.00	1.00
Male	1.33 (1.32-1.35)	1.37 (1.35-1.38)	1.35 (1.28-1.42)	1.42 (1.37-1.48)
**Carstairs deprivation index quintile**				
1 (least deprived)	1.00	1.00	1.00	1.00
2	1.07 (1.04-1.10)	1.04 (1.03-1.05)	1.02 (0.93-1.13)	1.04 (0.99-1.10)
3	1.23 (1.21-1.26)	1.13 (1.12-1.15)	1.23 (1.13-1.34)	1.12 (1.07-1.18)
4	1.30 (1.27-1.33)	1.26 (1.25-1.28)	1.25 (1.15-1.36)	1.26 (1.20-1.32)
5 (most deprived)	1.38 (1.35-1.41)	1.36 (1.35-1.38)	1.43 (1.31-1.56)	1.48 (1.41-1.56)
**Ethnicity**				
Black	0.61 (0.53-0.69)	0.83 (0.81-0.85)	0.98 (0.67-1.43)	1.54 (1.43-1.65)
Other and mixed	0.66 (0.60-0.72)	0.75 (0.73-0.78)	0.76 (0.54-1.07)	1.06 (0.95-1.18)
South Asian	0.66 (0.61-0.72)	0.84 (0.82-0.86)	0.84 (0.63-1.11)	1.16 (1.09-1.25)
White	1.00	1.00	1.00	1.00
**Region**				
London	0.70 (0.68-0.73)	0.92 (0.91-0.92)	0.78 (0.68-0.88)	1.25 (1.20-1.30)
Other	1.00	1.00	1.00	1.00
**Urban Rural**				
Rural	0.88 (0.86-0.89)	0.89 (0.89-0.90)	0.82 (0.77-0.88)	0.83 (0.79-0.86)
Urban	1.00	1.00	1.00	1.00
**HEALTH BEHAVIOURS / INDICATORS**				
**Body Mass Index**				
<18.5 (Underweight)	3.28 (3.20-3.36)	3.61 (3.56-3.67)	3.20 (2.90-3.53)	3.63 (3.42-3.86)
18.5-<25 (Normal weight)	1.00	1.00	1.00	1.00
25-<30 (Overweight)	0.65 (0.64-0.67)	0.67 (0.66-0.67)	0.66 (0.61-0.70)	0.71 (0.68-0.74)
30-<35 (Obesity class I)	0.67 (0.66-0.69)	0.72 (0.71-0.73)	0.73 (0.67-0.79)	0.81 (0.77-0.85)
>=35 (Obesity class II plus)	0.96 (0.93-0.98)	1.05 (1.03-1.06)	1.01 (0.92-1.11)	1.22 (1.15-1.29)
**Smoking status**				
Current smoker	2.52 (2.48-2.57)	2.23 (2.20-2.26)	2.30 (2.12-2.50)	1.83 (1.73-1.94)
Ex-smoker	1.42 (1.40-1.44)	1.30 (1.28-1.31)	1.35 (1.27-1.43)	1.27 (1.22-1.33)
Non-smoker	1.00	1.00	1.00	1.00
**MORBIDITY**				
**Autoimmune condition**				
Lupus erythematosus	1.78 (1.62-1.95)	1.59 (1.51-1.68)	1.73 (1.19-2.53)	1.08 (0.84-1.38)
Psoriasis	1.19 (1.16-1.22)	1.13 (1.11-1.14)	1.24 (1.12-1.36)	1.18 (1.11-1.24)
Rheumatoid arthritis	1.50 (1.45-1.55)	1.50 (1.47-1.53)	1.52 (1.32-1.74)	1.54 (1.44-1.66)
**Cardiovascular disease**				
Cerebrovascular disease	1.94 (1.91-1.97)	2.00 (1.97-2.02)	2.00 (1.88-2.13)	2.14 (2.05-2.24)
Chronic heart disease	1.90 (1.87-1.92)	2.02 (2.00-2.04)	1.87 (1.77-1.98)	2.04 (1.96-2.13)
Hypertension	1.37 (1.35-1.39)	1.24 (1.23-1.25)	1.34 (1.26-1.42)	1.38 (1.33-1.43)
Venous thromboembolism	2.14 (2.10-2.19)	2.36 (2.32-2.39)	2.11 (1.95-2.28)	2.29 (2.17-2.42)
**Chronic respiratory disease**				
Asthma	1.04 (1.02-1.07)	1.12 (1.11-1.14)	0.98 (0.90-1.07)	1.13 (1.08-1.18)
Other	2.47 (2.44-2.51)	2.59 (2.56-2.62)	2.30 (2.15-2.45)	2.32 (2.21-2.44)
**Neurological conditions**				
Dementia	3.26 (3.21-3.32)	3.45 (3.41-3.49)	4.40 (4.14-4.69)	5.02 (4.81-5.25)
Learning disabilities	3.40 (3.20-3.63)	3.57 (3.45-3.70)	4.85 (3.92-5.99)	5.08 (4.53-5.69)
Other associated with respiratory infections	2.32 (2.26-2.38)	2.40 (2.36-2.43)	2.67 (2.42-2.94)	2.52 (2.39-2.66)
**Other comorbidity**				
Cancer (diagnosed in last year)	9.70 (9.50-9.90)	11.9 (11.7-12.1)	8.20 (7.50-8.97)	8.68 (7.99-9.44)
Chronic kidney disease	2.14 (2.11-2.17)	1.96 (1.94-1.98)	2.18 (2.06-2.30)	2.20 (2.10-2.29)
Diabetes	1.64 (1.62-1.67)	1.64 (1.62-1.65)	1.81 (1.71-1.92)	1.93 (1.86-2.01)
Multimorbidity	2.33 (2.29-2.36)	2.79 (2.75-2.83)	2.36 (2.23-2.51)	2.90 (2.74-3.06)
