# Supplementary material for: Factors associated with excess all-cause mortality in the first wave of the COVID-19 pandemic in the UK: A time series analysis using the Clinical Practice Research Datalink
Source: PLoS Med. 2022 Jan 6;19(1):e1003870. doi: 10.1371/journal.pmed.1003870 (PMC8735664; doi:10.1371/journal.pmed.1003870)
Supplement: S5 Table — CI, confidence interval; RR, rate ratio. (PDF) [file pmed.1003870.s013.pdf]

S5 Table: All-cause relative rates of death and 95% confidence intervals by morbidities, health and demographic factors pre-pandemic and during Wave 1 adjusted for age, sex, season and year with and without linked data

	Pre-pandemic		During Wave 1	
	**Primary care only**	**Linked data**	**Primary care only**	**Linked data**
**DEMOGRAPHICS**				
**Age**				
5 year increase in age	1.67 (1.67-1.68)	1.70 (1.69-1.70)	1.70 (1.69-1.71)	1.76 (1.74-1.78)
**Deprivation index* quintile**				
1 (least deprived)	1.00	1.00	1.00	1.00
2	1.04 (1.03-1.05)	1.04 (1.03-1.05)	1.05 (1.00-1.10)	1.03 (0.98-1.09)
3	1.16 (1.14-1.17)	1.14 (1.12-1.15)	1.15 (1.10-1.20)	1.12 (1.07-1.18)
4	1.28 (1.27-1.29)	1.27 (1.25-1.28)	1.26 (1.20-1.31)	1.26 (1.20-1.33)
5 (most deprived)	1.37 (1.35-1.38)	1.36 (1.34-1.37)	1.48 (1.42-1.55)	1.49 (1.42-1.56)
**Ethnicity**				
Black	0.80 (0.78-0.82)	0.76 (0.74-0.78)	1.50 (1.40-1.61)	1.48 (1.38-1.59)
Other and mixed	0.74 (0.71-0.76)	0.71 (0.69-0.74)	1.02 (0.92-1.13)	1.05 (0.94-1.16)
South Asian	0.82 (0.80-0.83)	0.74 (0.72-0.75)	1.13 (1.06-1.21)	1.12 (1.05-1.19)
White	1.00	1.00	1.00	1.00
**Region**				
London	0.89 (0.88-0.89)	0.89 (0.88-0.90)	1.20 (1.16-1.25)	1.23 (1.18-1.28)
Other	1.00	1.00	1.00	1.00
**Sex**				
Female	1.00	1.00	1.00	1.00
Male	1.36 (1.35-1.37)	1.36 (1.34-1.37)	1.42 (1.37-1.47)	1.37 (1.32-1.42)
**Urban Rural**				
Rural	0.89 (0.88-0.90)	0.90 (0.89-0.91)	0.82 (0.79-0.86)	0.84 (0.81-0.88)
Urban	1.00	1.00	1.00	1.00
**HEALTH BEHAVIOURS / INDICATORS**				
**Body Mass Index**				
<18.5 (Underweight)	3.67 (3.62-3.72)	3.61 (3.56-3.66)	3.67 (3.47-3.89)	3.60 (3.38-3.82)
18.5-<25 (Normal weight)	1.00	1.00	1.00	1.00
25-<30 (Overweight)	0.67 (0.66-0.67)	0.67 (0.66-0.68)	0.70 (0.67-0.73)	0.71 (0.67-0.74)
30-<35 (Obesity class I)	0.71 (0.70-0.72)	0.72 (0.71-0.73)	0.80 (0.76-0.84)	0.83 (0.78-0.87)
>=35 (Obesity class II plus)	1.03 (1.02-1.05)	1.06 (1.04-1.07)	1.18 (1.12-1.25)	1.24 (1.17-1.31)
**Smoking status**				
Current smoker	2.33 (2.30-2.36)	2.23 (2.20-2.25)	1.98 (1.88-2.09)	1.69 (1.60-1.79)
Ex-smoker	1.33 (1.32-1.35)	1.44 (1.42-1.45)	1.31 (1.25-1.36)	1.30 (1.25-1.36)
Non-smoker	1.00	1.00	1.00	1.00
**MEDICAL CONDITION**				
**Autoimmune condition**				
Lupus erythematosus	1.64 (1.56-1.72)	1.62 (1.54-1.71)	1.22 (0.99-1.50)	1.13 (0.88-1.45)
Psoriasis	1.14 (1.13-1.16)	1.13 (1.11-1.15)	1.19 (1.13-1.25)	1.17 (1.10-1.24)
Rheumatoid arthritis	1.50 (1.48-1.53)	1.50 (1.48-1.53)	1.55 (1.45-1.65)	1.56 (1.45-1.68)
**Cardiovascular disease**				
Cerebrovascular disease	2.02 (2.00-2.04)	1.99 (1.97-2.01)	2.15 (2.07-2.24)	2.19 (2.09-2.28)
Chronic heart disease	2.03 (2.01-2.05)	2.00 (1.98-2.02)	2.05 (1.98-2.13)	2.08 (2.00-2.17)
Hypertension	1.28 (1.27-1.29)	1.23 (1.21-1.24)	1.38 (1.33-1.43)	1.41 (1.36-1.46)
Venous thromboembolism	2.43 (2.39-2.46)	2.36 (2.33-2.39)	2.38 (2.26-2.51)	2.33 (2.20-2.46)
**Chronic respiratory disease**				
Asthma	1.11 (1.10-1.12)	1.12 (1.11-1.13)	1.11 (1.06-1.15)	1.14 (1.09-1.19)
Other	2.63 (2.60-2.66)	2.59 (2.56-2.63)	2.38 (2.27-2.49)	2.36 (2.24-2.48)
**Neurological conditions**				
Dementia	3.48 (3.44-3.51)	3.44 (3.40-3.48)	5.02 (4.82-5.23)	5.12 (4.90-5.34)
Learning disabilities	3.54 (3.43-3.65)	3.85 (3.73-3.98)	5.04 (4.56-5.58)	5.49 (4.93-6.10)
Other associated+	2.39 (2.36-2.42)	2.39 (2.35-2.42)	2.57 (2.45-2.70)	2.58 (2.45-2.73)
**Other comorbidity**				
Cancer (diagnosed in last year)	12.6 (12.4-12.8)	15.1 (14.8-15.4)	9.52 (8.76-10.34)	10.8 (10.0-11.8)
Chronic kidney disease	2.06 (2.03-2.08)	1.95 (1.93-1.97)	2.27 (2.18-2.36)	2.24 (2.15-2.35)
Diabetes	1.66 (1.64-1.68)	1.75 (1.73-1.76)	1.95 (1.88-2.02)	2.11 (2.02-2.20)
Multimorbidity	2.62 (2.59-2.65)	5.12 (5.05-5.19)	2.67 (2.54-2.80)	5.00 (4.71-5.31)
