# Supplementary material for: Factors associated with excess all-cause mortality in the first wave of the COVID-19 pandemic in the UK: A time series analysis using the Clinical Practice Research Datalink
Source: PLoS Med. 2022 Jan 6;19(1):e1003870. doi: 10.1371/journal.pmed.1003870 (PMC8735664; doi:10.1371/journal.pmed.1003870)
Supplement: S6 Table — CI, confidence interval; RR, rate ratio. (PDF) [file pmed.1003870.s014.pdf]

S6 Table. All cause-relative rates of death and 95% confidence intervals by ethnicity in the full study population, London, and the rest of the UK pre-pandemic and during Wave 1 adjusted for age, sex, season and year by age group

	**Before Wave 1**			**During Wave 1**		
	Overall	London only	Other	Overall	London only	Other
White	1.00	1.00	1.00	1.00	1.00	1.00
South Asian	0.82 (0.80-0.83)	0.81 (0.79-0.84)	0.84 (0.82-0.86)	1.13 (1.06-1.21)	1.10 (1.00-1.22)	1.03 (0.93-1.13)
Black	0.80 (0.78-0.82)	0.84 (0.82-0.87)	0.77 (0.73-0.80)	1.50 (1.40-1.61)	1.39 (1.27-1.52)	1.33 (1.17-1.52)
Other and mixed	0.74 (0.71-0.76)	0.71 (0.67-0.75)	0.78 (0.75-0.81)	1.02 (0.92-1.13)	1.06 (0.92-1.23)	0.86 (0.74-1.01)
Missing	1.13 (1.12-1.14)	0.96 (0.93-0.99)	1.13 (1.12-1.14)	1.04 (1.01-1.08)	0.97 (0.87-1.08)	1.05 (1.02-1.09)
