# Supplementary material for: Factors associated with excess all-cause mortality in the first wave of the COVID-19 pandemic in the UK: A time series analysis using the Clinical Practice Research Datalink
Source: PLoS Med. 2022 Jan 6;19(1):e1003870. doi: 10.1371/journal.pmed.1003870 (PMC8735664; doi:10.1371/journal.pmed.1003870)
Supplement: S7 Table — BMI, body mass index; CI, confidence interval; RR, rate ratio. (PDF) [file pmed.1003870.s015.pdf]

S7 Table. All cause-relative rates of death and 95% confidence intervals by ethnicity, Body Mass Index and Smoking status groups with a missing category pre-pandemic and during Wave 1 adjusted for age, sex, season and year by age group

	Before Wave 1	During Wave 1
Ethnicity		
Black	0.80 (0.78-0.82)	1.50 (1.40-1.61)
Other and mixed	0.74 (0.71-0.76)	1.02 (0.92-1.13)
South Asian	0.82 (0.80-0.83)	1.13 (1.06-1.21)
White	1.00	1.00
Missing	1.13 (1.12-1.14)	1.04 (1.01-1.08)
Body Mass Index		
<18.5 (Underweight)	3.67 (3.62-3.72)	3.67 (3.47-3.89)
18.5-<25 (Normal weight)	1.00	1.00
25-<30 (Overweight)	0.67 (0.66-0.67)	0.70 (0.67-0.73)
30-<35 (Obesity class I)	0.71 (0.70-0.72)	0.80 (0.76-0.84)
35-<40 (Obesity class II)	1.03 (1.02-1.05)	1.18 (1.12-1.25)
Missing	1.05 (1.03-1.06)	1.05 (0.99-1.11)
Smoking status		
Current smoker	2.33 (2.30-2.36)	1.98 (1.88-2.09)
Ex-smoker	1.33 (1.32-1.35)	1.31 (1.25-1.36)
Non-smoker	1.00	1.00
Missing	0.87 (0.85-0.88)	1.19 (1.10-1.29)
