# Supplementary material for: Factors associated with excess all-cause mortality in the first wave of the COVID-19 pandemic in the UK: A time series analysis using the Clinical Practice Research Datalink
Source: PLoS Med. 2022 Jan 6;19(1):e1003870. doi: 10.1371/journal.pmed.1003870 (PMC8735664; doi:10.1371/journal.pmed.1003870)
Supplement: Attachment — Submitted filename: Revisions letter Sept 2021 (submitted).docx [file pmed.1003870.s016.docx]

NOTES TO EDITORS AND REVIEWERS

Many thanks for your comments on our manuscript which have helped us to improve our description of the methodology and results and to extend our discussion. We have responded to your comments below and described the changes that we’ve made. **All line numbers are from the track changes version.**

**-----------------------------------------------------------
Requests from the editors:

At this stage, we ask that you include a short, non-technical Author Summary of your research to make findings accessible to a wide audience that includes both scientists and non-scientists. The Author Summary should immediately follow the Abstract in your revised manuscript. This text is subject to editorial change and should be distinct from the scientific abstract. Please see our author guidelines for more information:** [**https://journals.plos.org/plosmedicine/s/revising-your-manuscript#loc-author-summary**](https://protect-eu.mimecast.com/s/CCKRC14LACnVXYqTqJWRw?domain=journals.plos.org)

Author response: We have added a non-technical author summary as requested.

Author change:

Why Was This Study Done?

- All-cause mortality during the COVID-19 pandemic was substantially higher than in previous years, particularly among elderly people, males, people of non-white ethnicity, people of lower socio-economic status and people living in care-homes.
- There is little evidence of how pre-existing health and demographic factors are associated with all-cause mortality and how this compares to mortality risks in people with and without these factors before the pandemic.

What Did the Researchers Do and Find?

- We used seasonally adjusted time series models to estimate all-cause and excess mortality during Wave 1 of the UK pandemic (5^th^ March to 27^th^ May 2020) for people with individual health and socio-demographic factors.
- We then estimated relative risks of all-cause mortality in people with and without these factors before the pandemic and during Wave 1.
- We found similar relative risks of all-cause mortality in people with and without each factor before and during Wave 1 of the pandemic, but there were exceptions.
- Health-related exceptions were people with a diagnosis of dementia or learning disabilities, whose risk of death relative to people without these conditions was substantially higher during Wave 1 than it was before the pandemic.
- Socio-economic exceptions were non-white ethnicity and living in London; these groups had lower immediate risks of death before the pandemic compared to white people or people living outside of London, but substantially elevated relative risks during Wave 1.

What Do These Findings Mean?

- This suggests that COVID-19 has dialled up the risk of death by a similar proportional degree for most people.
- From a clinical perspective pre-pandemic knowledge about the relative frailty associated with different conditions can be reasonably applied in the pandemic situation.
- Exceptions include people with learning difficulties and dementia who may have been more exposed to infection in care homes and through personal care; and those of non-white ethnicity who may have been at higher risk due to increased social interactions.

**Please remove the ‘Authors and contributors’, ‘Data availability’, ‘Competing interests’, ‘Financial disclosure’, and ‘Open Access Statement’ from the end of the main text. In the event of publication, the information from the first four sections will be published as metadata based on your response to the submission form. All manuscripts published in PLOS Medicine are made available under the terms of a CC-BY licence, so there is no need for the Open Access Statement.**

Author response: We have removed these sections as requested.

**Lines 422-424: Rather than deleting this content, please relocate it to the Methods. Please note that, for the analytical code, the study author cannot be the contact person. We would recommend either providing the code in the supplementary materials, uploading it to a public repository, or (if necessary) using an alternative contact for requests.**

Author response:

Thank you. The study protocol is now mentioned in the methods.

We are adding all analytical code to our institution’s public data platform using the same web address as the code lists. This will be available within the next two weeks.

Author change (Methods line 127): We have moved the following sentence from the data availability section to the methods: The pre-defined study protocol is available in the Supplementary Appendix (p2-21).

Author change (Methods line 222): All analytical code is available at <https://doi.org/10.17037/DATA.00002269> ..

**Please ensure that the study is reported according to the STROBE guideline, and include the completed STROBE checklist as Supporting Information. Please add the following statement, or similar, to the Methods: "****This study is reported as per the Strengthening the Reporting of Observational Studies in Epidemiology (STROBE) guideline (S1 Checklist)."

The STROBE guideline can be found here:** [**http://www.equator-network.org/reporting-guidelines/strobe/**](http://protect-eu.mimecast.com/s/aqyDC2WMBTKMQw8hQXLUs?domain=equator-network.org) **When completing the checklist, please use section and paragraph numbers, rather than page numbers.**

Author response: We have completed the Record checklist and added it to Supplementary appendix p35. We also amended our objectives to comply with the protocol.

Author change:

Added (Methods, line 128): The study is reported as per the Reporting of studies Conducted using Observational Routinely-collected health Data (RECORD) Statement[19] which is an extension of the Strengthening the Reporting of Observational Studies in Epidemiology (STROBE) guideline.

Changed (Introduction, line 119):

Before: We examined excess mortality in the UK during Wave 1 of the COVID-19 pandemic according to an extensive range of morbidities (including dementia) and socio-demographic characteristics. To do this we used a large regularly updated primary care database covering 20% of the UK population.

After: We estimated excess mortality in the UK during Wave 1 of the COVID-19 pandemic in people with an extensive range of morbidities (including dementia) and socio-demographic characteristics. We then estimated and compared relative risks of all-cause mortality in people with and without these factors before the pandemic and during Wave 1. To do this we used a large regularly updated primary care database covering 20% of the UK population.

**Comments from the reviewers:

Reviewer #1: Thank you for the opportunity to review the manuscript titled "Factors associated with excess all-cause mortality in the first wave of COVID-19 pandemic in the UK: a time-series analysis using the Clinical Practice Research Datalink."

The authors use electronic health records (EHR) data from primary care practices in the UK to examine factors associated with excess all-cause mortality during the first wave of the COVID-19 pandemic (March 5-May 27, 2020) among individuals 40 years or older. These data cover about 20% of the UK population and come from two different EHR data systems. Using pre-pandemic period from March 5, 2015 through March 4, 2020, the authors predict expected number of deaths overall and by subpopulations. They examine pre-existing health status (BMI, smoking status, and various morbidities), demographic factors (5-year age groups, sex, ethnicity), and other factors (area-level deprivation, region).

The authors find that during the first wave of the pandemic mortality risk increased by the same relative degree for most subpopulations examined (in other words, the observed differences in relative mortality rates between the pre-pandemic period and the first wave of the pandemic were very small for most groups). But disproportional increases in mortality were observed for patients with dementia, learning disabilities, non-white ethnicity, and living in London. The main findings are based on the model adjusted for age and sex.

This is a well-written paper using great data to examine all-cause excess mortality by various demographic factors and underlying health conditions. The data has many advantages. For example, it allows the authors to calculate numerators and denominators for the same population from the same data source. The authors are upfront about the data's limitations and offer a great set of robustness tests. For example, I found the robustness check to an alternative source of death data and to the inclusion of hospital diagnoses in addition to primary care ones very convincing. It addressed my initial concern about the process by which primary care practices get notified of deaths (if deaths happened in hospitals or nursing homes) and whether there were any lags in reporting this information.

The authors state that their data are representative of the UK population in terms of age and sex but I also wondered if the data are representative of the underlying health conditions and geographic distribution (since we know that the direct and indirect effects of the virus did not impact all geographic areas equally during the first wave of the pandemic). And, also whether there are differences in representativeness across the two data systems. Bias in EHR data is something I generally worry about as it is not clear how representative these patients are of all patients and of all people in the community. The authors acknowledged it and presented the findings separately by the data system which, again, I found convincing.

I would have preferred to see more discussion of related work, more citations to relevant literature and better way to highlight their contribution.**

Author response:

Thank you for your kind comments on our work. We added to our discussion the comparison with other relevant studies recently published. We have also added a non-technical author summary as requested by the editors.

Author change:

See non-technical author summary (above) and responses to comments 4 and 5.

**1) It would be useful to see the estimation equation in addition to the description provided in the paper. The methods are described in words but seeing the actual equation would answer many of my questions. For example, I was not entirely sure what the unit of observation was in any given estimation. The authors present excess deaths per million patient-weeks. Why not in terms of million patients?**

Author response: Many thanks for your suggestion. We have included the estimation equation in the supplementary appendix (see below). The dependent variable is deaths (count) and the unit of observation weeks. The model is offset by the denominator (i.e. number of patients) which varies for each week as patients enter and leave the cohort (i.e. by joining or leaving the practice, dying or having a change in health status recorded). We do not therefore have a static denominator that would allow us to estimate excess deaths per million patients.

Author change:

Added (Methods, line 182): The estimation equation for the basic model is included in Supplementary Appendix p24.

Added (Supplementary Appendix p24):

log(µ) = β_0_ + β _1_(year - 2017) + β _2_([year-2017]^2) + β_3_(sin[pi x degree/180]) + β_4_ (sin[2 x pi x degree/180]) + β_5_(sin[3 x pi x degree/180]) + β_6_(cos[pi x degree/180]) + β_7_ (cos[2 x pi x degree/180]) + β_8_ (cos[3 x pi x degree/180])

Var(µ)= µ+ α µ^2^

Where the unit of observation is in weeks:

µ=E(y/N)

y = deaths during the unit of observation (count)

N= number of patients during the unit of observation

degree = (week/52)*360

**2) It may be interesting to see the results showing remaining ethnicity gaps when controlling for underlying health conditions in addition to age and sex.**

Author response: We have used time series analyses to describe changes in relative mortality rates for people with different health and demographic characteristics during Wave 1 of the pandemic. Studying the extent of the ethnicity gap that would remain after removing the impact of underlying health condition would call upon causal inference methods. These would require invoking strong assumptions of no unmeasured confounding and methods that are beyond the scope of this paper. We are currently considering how to address this gap as described in the protocol (Supplementary appendix p2). We have added this as a topic for future research in our discussion.

Author change (Discussion, line 472)

Before: Future research may clarify whether there were differences across waves in the UK.

After: Future research may clarify whether there were differences across waves in the UK, particularly for factors such as ethnicity and deprivation, and investigate independent effects of individual health and demographic risk factors (e.g. ethnicity and health status).

**3) The paper's findings on dementia are consistent with existing literature finding that the pandemic disproportionally affected nursing home/care home residents. Risk of dementia increases with age and varies by race/ethnicity and preexisting health conditions. It would be interesting to see the dementia results when controlling for presence of other preexisting health conditions in addition to controlling for age/sex.**

Author response: Please see response to point 2.

**4) I would also encourage the authors to offer some thoughts on possible mechanisms. For example, most dementia patients receive care from other people to help them with their daily activities (so require close contact with people which increases the probability of infection). And, I suggest they cite related work specifically focusing on dementia patients. For example, Wang Q.Q., Davis P.B., Gurney M.E., Xu R. "COVID-19 and dementia: Analyses of risk, disparity, and outcomes from electronic health records in the US," 2021.**

Author response

Thank you. We expanded on the possible mechanisms and added the reference.

Author change (Discussion line 420):

Before: This disproportionate exposure to SARS-CoV-2 may explain the increased mortality rate ratio in these two groups of the population.

After:

This disproportionate exposure to SARS-CoV-2 may explain the increased mortality rate ratio in these two groups of the population, and is consistent with studies conducted elsewhere that showed increased risk of infection among people with dementia [41]. However, we cannot rule out that factors other than infection may explain the increased risk of mortality. Joy et al. [42] quantified risk of mortality in a cohort of people with known SARS-CoV-2 infection status, and found a similarly increased risk of mortality among people with learning disabilities and, compared to similar people without learning disabilities, suggesting that comorbidity and treatment may also explain part of the increased risk of mortality.

**5) While the authors highlight dementia and learning disability results, there is also significant evidence of an increase in racial/ethnic disparities which is consistent with other studies and I think this finding is also worth mentioning. Additionally, while small in magnitude, there is also an interesting finding that the most disadvantaged areas had a statistically significant increase in relative risk. This finding would need to be explored in future research to understand the causes behind this increase and examine whether this increase persisted as the pandemic continued.**

Author response

We agree with the reviewer and expanded on our results on ethnic disparities and socio-economic inequalities.

Author change (Added, Discussion line 447): Similar to other studies[16,42], the increase in the RR of mortality was disproportionately increased amongst those living in the most deprived areas, reflecting the perpetuation of the adverse effect of low socio-economic status in health and mortality.

Author change (Discussion, line 472)

Before: Future research may clarify whether there were differences across waves in the UK.

After: Future research may clarify whether there were differences across waves in the UK, particularly for factors such as ethnicity and deprivation, and investigate independent effects of individual health and demographic risk factors (e.g. ethnicity and health status).

**Minor points:
1) Suggest including the entire pre-period to Figure 2 so it is easy to see how well the prediction performed.**

Author response/change: We have added a graph for the study population as a whole to the Supplementary appendix page 25 which displays observed and predicted deaths per million person-weeks for the full study period for each comorbidity.

**2) Figure 3 does not include rural/urban results. Also, would it be possible to include all 5-year age categories instead of having just one 5-year age increase? In Figure 3, is everything adjusted for sex and age (including the 5-year increase in age and male categories)?**

Author response: Figure 3 includes rural/urban results under the demographics subheading. We decided not shown each 5-year age category to avoid overcrowding figure 3 and because the patterns are consistent for each age category. All models were adjusted for both age and sex except age which was adjusted for sex only and sex which was adjusted for age only.

Author change:

Added (Results line 303): Relative rates of death for each 5-year age category before and during Wave 1 are displayed in Supplementary Appendix p27.

Added (Supplementary Appendix p27):

All-cause relative rates of death and 95% confidence intervals by 5-year age category pre-pandemic and during Wave 1 adjusted for sex, season and year

**3) I was not sure if the post-period (May 28-July 31, 2020) is included in any of the analysis. Figure 2 does not show any excess deaths in July.**

Author response: The post-period (May 28-July 31, 2020) is shown for illustrative purposes only. We have made the changes described below to clarify this.

change excess deaths per million person weeks to excess deaths per million person weeks in Wave 1

Author change (figure 2 text box)

Before: excess deaths per million person weeks

After: excess deaths in Wave 1 per million person weeks

Author change (figure 2 legend, p11 line 269)

Added: Dotted red lines – Start and end of Wave 1

**4) Is the deprivation measure based on patient or practice location?**

Author response: The deprivation measure is based on the practice location for the primary analysis because this is available for all patients. In the secondary analysis, we used patient level deprivation status. This is described as follows in line 141. “CPRD primary care data may be linked to additional health and area-based deprivation data sets. Quintiles of the Carstairs Index and urban-rural data were added by CPRD through linkage using the practice postcode[21]. Linkages based on patient data are also available[22], but are restricted to English practices that have agreed to participate in the linkage programme, reducing geographic coverage and the sample size. In sensitivity analyses, we used the Office for National Statistics (ONS) mortality data (national death registration data), the ICD-10 coded clinical data from Hospital Episode Statistics Admitted Patient Care (HES APC)[23], and patient-level quintile of Carstairs Index.”

The relative rates from primary care only compared to linked data are presented in supplementary appendix p32; they are almost identical.

**Reviewer #2: "Factors associated with excess all-cause mortality in the first wave of COVID-19 pandemic in the UK: a time-series analysis using the Clinical Practice Research Datalink" explores excess mortality in the UK from a largely-representative dataset covering some 20% of the domestic population, with a focus on the first wave of the pandemic (defined as 5th March to 27th May 2020), against expected mortality as modelled on data from 2015 onwards. The relative rate of death (RR) was computed for a large number of health and demographic factors (Figure 3), adjusted for age and sex where appropriate. An overall adjusted rate ratio of 1.43 was found for the first wave, broadly consistent with prior work on the topic, with the observation that "the net effect of COVID-19 in different sub-groups of the population is to simply amplify baseline mortality risk by a constant amount" (Line 378), though significant change in RR could be observed for some factors (e.g. dementia,**
**learning disabilities).**

**The analysis is generally comprehensive and convincing. However, some points might be clarified, although they would likely not materially affect the main findings:**

**1. For observed vs. modelled mortality (covered in the Statistical Analysis section and Figure 2), it would be highly recommended to provide more details about the accuracy of the model in fitting known data (apparently represented as solid grey lines in Figure 2, and currently only "visually checked" from Line 143). This is because the modelled mortality underpins the entire analysis. For example, modelled vs. actual (weekly) mortality might be plotted for each of the years 2015-2019 in a (supplementary) figure, and some quantitative estimate of any deviance between modelled and actual mortality might be made, for these years.**

Author response: We presented graphs of observed and predicted deaths per million person-weeks for each comorbidity in supplementary appendix page 25. We have added a graph for the study population as a whole and further analysis of deviance residuals.

Author change (Methods line 188):

Before: We visually checked the adequacy of our generalised linear models by comparing observed and expected deaths in the pre-pandemic period.

After: We checked the adequacy of our generalised linear models by comparing observed and expected deaths in the pre-pandemic period; we additionally assessed the distribution of the deviance residuals and estimated mean weekly deviance residuals as a proportion of weekly death counts.

Author change (Results line 250):

 Before: Patterns of observed and predicted deaths taking annual and seasonal patterns into account for each comorbidity are described in Supplementary Appendix p25. Model fit was good for most covariates. Seasonal patterns were consistent, except for people with recent cancer diagnoses where they were less pronounced.

After: Patterns of observed and predicted deaths for the full study period for each comorbidity, taking annual and seasonal patterns into account, are described in Supplementary Appendix p25; mean (sd) deviance residuals are tabulated in Supplementary Appendix p26. Model fit was good for most covariates; deviance residuals were near-normally distributed with few outliers. Mean absolute deviance as a proportion of weekly deaths was low for the study population (mean 0.04 sd 0.03) and highest for comorbidities with lower death counts such as learning difficulties (mean 0.24 sd 0.27). Seasonal patterns were consistent, except for people with recent cancer diagnoses where they were less pronounced. Mortality rates peaked in the severe 2017/2018 influenza season and decreased gradually in subsequent years.

Added (Supplementary Appendix p26)

Distribution of deviance residuals in the pre-pandemic period for the basic model

| **Factor** | **Mean weekly deaths (sd)** | **Mean weekly absolute deviance (sd)** | **Mean (sd) weekly absolute deviance as a proportion of weekly deaths** |
| --- | --- | --- | --- |
| Study population | 2,042.4 (235.9) | 86.1 (77.7) | 0.04 (0.03) |
| Hypertension | 1,461.9 (177.1) | 64.9 (58.2) | 0.04 (0.03) |
| Chronic kidney disease | 1,143.1 (144.8) | 53.1 (47.5) | 0.05 (0.04) |
| Chronic heart disease | 694.5 (88.5) | 35.8 (29.4) | 0.05 (0.04) |
| Diabetes | 493.2 (65.1) | 26.9 (21.5) | 0.05 (0.04) |
| Dementia | 441.0 (77.4) | 30.1 (26.5) | 0.07 (0.05) |
| Other respiratory | 435.7 (64.1) | 24.5 (20.3) | 0.06 (0.04) |
| Multimorbidity | 399.1 (54.9) | 22.1 (17.4) | 0.06 (0.04) |
| Cerebrovascular disease | 397.4 (50.5) | 22.7 (17.8) | 0.06 (0.04) |
| Venous thromboembolism | 218.5 (30.5) | 14.9 (11.0) | 0.07 (0.05) |
| Cancer (diagnosed in last year) | 209.9 (16.8) | 12.3 (9.9) | 0.06 (0.05) |
| Asthma | 190.1 (28.8) | 13.8 (10.9) | 0.07 (0.06) |
| Other neurological | 124.6 (19.2) | 10.7 (8.1) | 0.09 (0.07) |
| Psoriasis | 106.1 (16.1) | 9.1 (6.6) | 0.09 (0.06) |
| Rheumatoid arthritis | 58.8 (10.1) | 6.3 (4.8) | 0.11 (0.09) |
| Learning disabilities | 15.8 (4.9) | 3.4 (2.6) | 0.24 (0.27) |

**2. Further on Figure 2, it is recommended to indicate the specific years (2015-2019) for the grey lines if possible, perhaps by using line break patterns/colours. Moreover, it appears that the predicted mortality per million (i.e. assuming no pandemic) for 2020, is lower than the previous years; is this an expected continuation of an existing pattern, and if so, what might be the cause (e.g. changing demographics due to immigration?). This might be briefly discussed.**

Author response: Many thanks for your suggestion. We implemented this but the lines are close together and overlapping which makes the different line patterns difficult to interpret. As mentioned above, we have added a study population graph to supplementary appendix page 25 which describes observed and predicted deaths per million person-weeks for the whole study period. A pattern of decreasing deaths was observed from 2018 to 2019 following a severe flu season in 2017/2018; our model predicted a further decrease in number of deaths per million person years in 2020. Nevertheless, we observed lower than expected mortality rates in January and February 2020 (i.e. before the pandemic).

Author change (added to Results line 258): Mortality rates peaked in the severe 2017/2018 influenza season and decreased gradually in subsequent years.

**3. While a model is required to predict the mortality for 2020 had there been no pandemic, it is not as clear as to why the model had to be fitted by splines to the observed (real) deaths in 2020 (solid red line, Line 208). Might it not be desirable to calculate excess mortality by subtracting (modelled) predicted mortality from (actual) observed mortality, instead of subtracting it from (fitted) mortality? This might be explained further, and if possible a brief sensitivity analysis on actual observed mortality might be considered.**

Author response: We fitted the spline model for illustrative purposes only. Excess mortality was calculated directly from the observed results as you suggest. We have modified the figure legend to make this clearer.

Author change (figure 2 legend, line 271)

Before: Excess deaths per million patient weeks = Total number of observed deaths in Wave 1 of the pandemic minus predicted number according to the 2015-2019 model

After: Excess deaths per million patient weeks = Total number of observed deaths (grey dots) in Wave 1 of the pandemic minus predicted number according to the 2015-2019 model (solid black line)

**4.** **The fitting for the basic generalized linear models as described in Lines 134 to 146 might be expounded upon in greater detail, possibly in supplementary material, with design choices discussed (e.g. [hyper]parameters chosen for Fourier terms/the quadratic function) with relevant references cited in support, if appropriate (there appears currently only a brief mention of a BMJ 2016 paper, in Page 17 of the supplementary material).**

Author response: We based our general approach to adjusting for seasonality and year on year trends on methods described by Bhaskaran et al*. We found that using 3 pairs of Fourier terms modelled seasonal trends effectively without overfitting and that adding a quadratic term for year (and centering year) captured a degree of non-linearity in the year-on-year trends whilst avoiding overfitting with higher order polynomials.

*Bhaskaran K, Gasparrini A, Hajat S, Smeeth L, Armstrong B. Time series regression studies in environmental epidemiology. Int J Epidemiol. 2013;42: 1187–1195. doi:10.1093/ije/dyt092

We have added this reference to our description of the model and provided more information about the model specification and fit (see responses to your first comment and reviewer 1 comment 1).

Author change (Methods, line 178)

Before: All models included Fourier terms (for creating a harmonic function of calendar week) to capture seasonality in death rates, and a quadratic function of calendar year (treated as a continuous variable) to capture any major underlying trends.

After: All models included 3 pairs of Fourier terms (for creating a harmonic function of calendar week) to capture seasonality in death rates; 3 pairs of terms were chosen as we wished to adequately capture some degree of complexity in seasonal patterns without overfitting. A quadratic function of calendar year (treated as a continuous variable) was used to capture any major underlying trends allowing for non-linearity[28].

**5. If possible, similar details might be added for the secondary analyses. For example, for the cohort approach, it is currently stated that "Interactions between the pandemic indicator and covariates will be added one at a time initially and in combinations, where possible, to describe differences in excess mortality between risk factor groups" - is there any protocol/method behind the order/combinations of such interactions?**

Author response: The above text is from the full protocol which is included in the supplementary appendix. As described in the “Changes to approved protocol” on p20, this manuscript is based on the primary time series analysis only. We have added the need for further research investigating independent associations to the discussion.

Author change (Methods, line 472)

Before: Future research may clarify whether there were differences across waves in the UK.

After: Future research may clarify whether there were differences across waves in the UK, particularly for factors such as ethnicity and deprivation, and investigate independent effects of individual health and demographic risk factors (e.g. ethnicity and health status).

**6. The overall excess mortality found might be briefly discussed against prior works on aggregate country-level excess mortality, and not merely excess mortality by health/demographic factors; although the analysis in this manuscript involves only the population aged 40 and above, such comparisons should still be reasonable given that mortality amongst the younger population is relatively negligible.**

Author response: We observed an overall relative rate of death in Wave 1 compared to the pre-pandemic period of 1.43 (95% CI 1.40-1.47), following adjustment for seasonality, year, age, and sex. This compares closely to a 47% increase in death observed in England and Wales during the same period in ONS mortality data compared to expected deaths based on an average from 2015-1019.

Author change (Discussion line 400): Our observation of an overall relative rate of death in Wave 1 compared to the pre-pandemic period of 1.43 (95% CI 1.40-1.47), following adjustment for seasonality, year, age, and sex, compares closely to a 47% increase in death observed in England and Wales during the same period in ONS mortality data compared to expected deaths based on an average from 2015-1019 (estimated from data provided by the ONS[35,36]).

**Reviewer #3:**

**1.Line 143: What do the authors mean by saying We visually checked the adequacy of our generalised linear models by comparing observed and expected deaths in the pre-pandemic period?**

Author response: We presented graphs of observed and predicted deaths per million person-weeks for each comorbidity in supplementary appendix page 25 and visually compared differences between observed and modelled deaths in the pre-pandemic period. We have extended our analyses of model fit in response to reviewer 2 (comment 1).

Author change: See reviewer 2 (comment 1).

**2.Is the negative binomial model an hierarchical model? If not, why not?**

Author response: The negative binomial model that we used was not hierarchical in the sense of accounting for clustering within subsets. In some studies of individual-level data, clustering by data collection sites such as clinics or hospitals can be important due to differing practices or data recording between sites. However, this was not appropriate for the present study, which was ecological rather than at the individual-level; furthermore, our outcome was mortality and we do not think there is likely to be important clustering in recording of deaths. Another form of clustering that the reviewer may be alluding to here is autocorrelation, which is specifically relevant to time-series studies; this is when there are correlations between sequential observations due to the nature of the data (which reflect smooth changes over time). Autocorrelation in the raw data is generally greatly reduced by accounting for long-term trends and seasonality. As described in the manuscript, we generated partial autocorrelation plots for the final season/trend-adjusted models to check for this, and found no evidence of residual autocorrelation, thus no further adjustment was deemed necessary.

**3. Were GP practices in the study subject to data quality exclusions re reporting of outcomes and risk factors.**

Author response:

We have used CPRD’s patient level acceptable flag to restrict our cohort to patients who were permanently registered in the CPRD practice and had logical administrative date (e.g. registered on or after date of birth). CPRD also provide a practice level up to standard date for CPRD GOLD data which indicates when a practice started to collect detailed electronic health record date. We have not used this date for our study because our study period started in 2015 (i.e. long after the universal use of electronic health record systems in primary care).

We acknowledge the potential for misclassification of outcomes and risk factors and have discussed this in our limitations section. The validity of our outcome and risk factor definitions are supported by published validation studies and our sensitivity analysis using ONS mortality data in place of CPRD’s primary care derived death date, and linked Hospital Episode Statistics data to supplement our comorbidities definition. This gives us confidence that the practices included in our study provided good quality data despite potential variation between them which we’ve now highlighted.

Author change (Discussion, line 376, added text in green)

Added text in bold green: There is also a potential for misclassification of the exposures, as information may be incomplete (e.g. diagnoses from secondary care not coded in the primary care record), or inaccurate (e.g. patients correctly reporting their smoking behaviour), **and vary between practices.**

**4. The observed and predicted deaths in the learning disabilities group seem to show much greater variation (in non pandemic period) than for other groups. Given that one of the conclusions of the study concerns the impact of COVID-19 on this group why did the authors not seek to diagnose miss-specification, rather than eyeball obs-v-exp?**

Author response: We have described our approach to model checking in response to point 1 above. Learning disabilities affect a smaller and younger population than other comorbidities. The higher proportional deviance for learning disabilities is therefore due to low death counts over time which results in greater chance variation. Nevertheless, the observed and predicted patterns are similar to other variables and there is a clear observed increase in the mortality rate during the pandemic.

Author change: See comment 1. This includes the sentence “Mean absolute deviance as a proportion of weekly deaths was low for the study population (mean 0.04 sd 0.03) and highest for comorbidities with lower death counts such as learning difficulties (mean 0.24 sd 0.27).”

**5. Line 101: Other studies have shown considerable variation in excess deaths across geographical areas much smaller than NHS Region (ref 13 in m/s). Given post-code, smaller geographical areas could have been studied, such regional variation may impact on the main findings. The authors should consider discussing this, in particular the variation across smaller geographical scales.**

Author response: We were not able to study variation across smaller geographical scales in this study as these data are not provided by CPRD. This does not affect the internal validity of our study as we included patients from the same practices before and during the pandemic and have not adjusted for region in our regression models. These data may have been a useful resource to further explore differences that we observed in our RRs for ethnicity and region when we separated the CPRD GOLD and CPRD Aurum databases. We have extended our comments about this in the discussion:

Author change (Discussion line 385):

Before: Even though CPRD data are representative of the UK population in terms of age and sex, they include few practices from Eastern England [22]. This may explain differences observed in RRs for ethnicity and region in our sensitivity analyses separating the CPRD GOLD and CPRD Aurum databases. For analyses of health factors, we see no reasons to doubt that the pattern observed elsewhere in the UK would not be applicable.

After: Even though CPRD data are representative of the UK population in terms of age and sex, they are not regionally representative and include few practices from Eastern England[20]. This, together with likely clustering of practices within regions by Clinical Commissioning Group (CCG), may explain differences observed in RRs for ethnicity and region in our sensitivity analyses separating the CPRD GOLD and CPRD Aurum databases. For analyses of health factors, we see no reasons to doubt that the pattern observed elsewhere in the UK would not be applicable.

**6. Also, is it not possible to resolve the urban-rural classification further, eg to include conurbation etc**

Author response: We agree that it would be interesting to resolve the urban-rural classification. However, CPRD only provide the binary variable.
