# Supplementary material for: Factors associated with excess all-cause mortality in the first wave of the COVID-19 pandemic in the UK: A time series analysis using the Clinical Practice Research Datalink
Source: PLoS Med. 2022 Jan 6;19(1):e1003870. doi: 10.1371/journal.pmed.1003870 (PMC8735664; doi:10.1371/journal.pmed.1003870)
Supplement: Peer review history

# Editor report: Decision Letter 0

**DOI:** 10.1371/journal.pmed.1003870.r001

**Authors:** Callam Davidson

17 Jun 2021

Dear Dr Strongman, 

Thank you for submitting your manuscript entitled "Factors associated with excess all-cause mortality in the first wave of COVID-19 pandemic in the UK: a time-series analysis using the Clinical Practice Research Datalink" for consideration by PLOS Medicine.

Your manuscript has now been evaluated by the PLOS Medicine editorial staff and I am writing to let you know that we would like to send your submission out for external peer review.

However, before we can send your manuscript to reviewers, we need you to complete your submission by providing the metadata that is required for full assessment. To this end, please login to Editorial Manager where you will find the paper in the 'Submissions Needing Revisions' folder on your homepage. Please click 'Revise Submission' from the Action Links and complete all additional questions in the submission questionnaire.

Please re-submit your manuscript within two working days, i.e. by Jun 21 2021 11:59PM.

Login to Editorial Manager here: https://www.editorialmanager.com/pmedicine

Once your full submission is complete, your paper will undergo a series of checks in preparation for peer review. Once your manuscript has passed all checks it will be sent out for review. 

Feel free to email us at plosmedicine@plos.org if you have any queries relating to your submission.

Kind regards,

Callam Davidson

Associate Editor

PLOS Medicine

# Peer review: Decision Letter 1

**DOI:** 10.1371/journal.pmed.1003870.r002

**Authors:** Callam Davidson

1 Sep 2021

Dear Dr. Strongman,

Thank you very much for submitting your manuscript "Factors associated with excess all-cause mortality in the first wave of COVID-19 pandemic in the UK: a time-series analysis using the Clinical Practice Research Datalink" (PMEDICINE-D-21-02569R1) for consideration at PLOS Medicine. 

Your paper was evaluated by a senior editor and discussed among all the editors here. It was also discussed with an academic editor with relevant expertise, and sent to independent reviewers, including a statistical reviewer. The reviews are appended at the bottom of this email and any accompanying reviewer attachments can be seen via the link below:

[LINK]

In light of these reviews, we will not be able to accept the manuscript for publication in the journal in its current form, but we would like to consider a revised version that addresses the reviewers' and editors' comments. We cannot make any decision about publication until we have seen the revised manuscript and your response, and we plan to seek re-review by one or more of the reviewers. 

In revising the manuscript for further consideration, your revisions should address the specific points made by each reviewer and the editors. Please also check the guidelines for revised papers at http://journals.plos.org/plosmedicine/s/revising-your-manuscript for any that apply to your paper. In your rebuttal letter you should indicate your response to the reviewers' and editors' comments, the changes you have made in the manuscript, and include either an excerpt of the revised text or the location (eg: page and line number) where each change can be found. Please submit a clean version of the paper as the main article file; a version with changes marked should be uploaded as a marked up manuscript.

In addition, we request that you upload any figures associated with your paper as individual TIF or EPS files with 300dpi resolution at resubmission; please read our figure guidelines for more information on our requirements: http://journals.plos.org/plosmedicine/s/figures. While revising your submission, please upload your figure files to the PACE digital diagnostic tool, https://pacev2.apexcovantage.com/. PACE helps ensure that figures meet PLOS requirements. To use PACE, you must first register as a user. Then, login and navigate to the UPLOAD tab, where you will find detailed instructions on how to use the tool. If you encounter any issues or have any questions when using PACE, please email us at PLOSMedicine@plos.org.

We hope to receive your revised manuscript by Sep 22 2021 11:59PM. Please email us (plosmedicine@plos.org) if you have any questions or concerns.

***Please note while forming your response, if your article is accepted, you may have the opportunity to make the peer review history publicly available. The record will include editor decision letters (with reviews) and your responses to reviewer comments. If eligible, we will contact you to opt in or out.***

We ask every co-author listed on the manuscript to fill in a contributing author statement, making sure to declare all competing interests. If any of the co-authors have not filled in the statement, we will remind them to do so when the paper is revised. If all statements are not completed in a timely fashion this could hold up the re-review process. If new competing interests are declared later in the revision process, this may also hold up the submission. Should there be a problem getting one of your co-authors to fill in a statement we will be in contact. YOU MUST NOT ADD OR REMOVE AUTHORS UNLESS YOU HAVE ALERTED THE EDITOR HANDLING THE MANUSCRIPT TO THE CHANGE AND THEY SPECIFICALLY HAVE AGREED TO IT. You can see our competing interests policy here: http://journals.plos.org/plosmedicine/s/competing-interests.

Please use the following link to submit the revised manuscript: 

https://www.editorialmanager.com/pmedicine/

Your article can be found in the "Submissions Needing Revision" folder. 

To enhance the reproducibility of your results, we recommend that you deposit your laboratory protocols in protocols.io, where a protocol can be assigned its own identifier (DOI) such that it can be cited independently in the future. Additionally, PLOS ONE offers an option to publish peer-reviewed clinical study protocols. Read more information on sharing protocols at https://plos.org/protocols?utm_medium=editorial-email&utm_source=authorletters&utm_campaign=protocols

Please ensure that the paper adheres to the PLOS Data Availability Policy (see http://journals.plos.org/plosmedicine/s/data-availability), which requires that all data underlying the study's findings be provided in a repository or as Supporting Information. For data residing with a third party, authors are required to provide instructions with contact information for obtaining the data. PLOS journals do not allow statements supported by "data not shown" or "unpublished results." For such statements, authors must provide supporting data or cite public sources that include it. 

Thank you for your patience during the review process, we look forward to receiving your revised manuscript. If you have any questions, please don't hesitate to contact me. 

Sincerely,

Callam Davidson, 

Associate Editor

PLOS Medicine

cdavidson@plos.org

-----------------------------------------------------------

Requests from the editors:

At this stage, we ask that you include a short, non-technical Author Summary of your research to make findings accessible to a wide audience that includes both scientists and non-scientists. The Author Summary should immediately follow the Abstract in your revised manuscript. This text is subject to editorial change and should be distinct from the scientific abstract. Please see our author guidelines for more information: https://journals.plos.org/plosmedicine/s/revising-your-manuscript#loc-author-summary

Please remove the ‘Authors and contributors’, ‘Data availability’, ‘Competing interests’, ‘Financial disclosure’, and ‘Open Access Statement’ from the end of the main text. In the event of publication, the information from the first four sections will be published as metadata based on your response to the submission form. All manuscripts published in PLOS Medicine are made available under the terms of a CC-BY licence, so there is no need for the Open Access Statement.

Lines 422-424: Rather than deleting this content, please relocate it to the Methods. Please note that, for the analytical code, the study author cannot be the contact person. We would recommend either providing the code in the supplementary materials, uploading it to a public repository, or (if necessary) using an alternative contact for requests. 

Please ensure that the study is reported according to the STROBE guideline, and include the completed STROBE checklist as Supporting Information. Please add the following statement, or similar, to the Methods: "This study is reported as per the Strengthening the Reporting of Observational Studies in Epidemiology (STROBE) guideline (S1 Checklist)."

The STROBE guideline can be found here: http://www.equator-network.org/reporting-guidelines/strobe/

When completing the checklist, please use section and paragraph numbers, rather than page numbers.

Comments from the reviewers:

Reviewer #1: Thank you for the opportunity to review the manuscript titled "Factors associated with excess all-cause mortality in the first wave of COVID-19 pandemic in the UK: a time-series analysis using the Clinical Practice Research Datalink." 

The authors use electronic health records (EHR) data from primary care practices in the UK to examine factors associated with excess all-cause mortality during the first wave of the COVID-19 pandemic (March 5-May 27, 2020) among individuals 40 years or older. These data cover about 20% of the UK population and come from two different EHR data systems. Using pre-pandemic period from March 5, 2015 through March 4, 2020, the authors predict expected number of deaths overall and by subpopulations. They examine pre-existing health status (BMI, smoking status, and various morbidities), demographic factors (5-year age groups, sex, ethnicity), and other factors (area-level deprivation, region).

The authors find that during the first wave of the pandemic mortality risk increased by the same relative degree for most subpopulations examined (in other words, the observed differences in relative mortality rates between the pre-pandemic period and the first wave of the pandemic were very small for most groups). But disproportional increases in mortality were observed for patients with dementia, learning disabilities, non-white ethnicity, and living in London. The main findings are based on the model adjusted for age and sex.

This is a well-written paper using great data to examine all-cause excess mortality by various demographic factors and underlying health conditions. The data has many advantages. For example, it allows the authors to calculate numerators and denominators for the same population from the same data source. The authors are upfront about the data's limitations and offer a great set of robustness tests. For example, I found the robustness check to an alternative source of death data and to the inclusion of hospital diagnoses in addition to primary care ones very convincing. It addressed my initial concern about the process by which primary care practices get notified of deaths (if deaths happened in hospitals or nursing homes) and whether there were any lags in reporting this information.

The authors state that their data are representative of the UK population in terms of age and sex but I also wondered if the data are representative of the underlying health conditions and geographic distribution (since we know that the direct and indirect effects of the virus did not impact all geographic areas equally during the first wave of the pandemic). And, also whether there are differences in representativeness across the two data systems. Bias in EHR data is something I generally worry about as it is not clear how representative these patients are of all patients and of all people in the community. The authors acknowledged it and presented the findings separately by the data system which, again, I found convincing.

I would have preferred to see more discussion of related work, more citations to relevant literature and better way to highlight their contribution. 

1) It would be useful to see the estimation equation in addition to the description provided in the paper. The methods are described in words but seeing the actual equation would answer many of my questions. For example, I was not entirely sure what the unit of observation was in any given estimation. The authors present excess deaths per million patient-weeks. Why not in terms of million patients?

2) It may be interesting to see the results showing remaining ethnicity gaps when controlling for underlying health conditions in addition to age and sex.

3) The paper's findings on dementia are consistent with existing literature finding that the pandemic disproportionally affected nursing home/care home residents. Risk of dementia increases with age and varies by race/ethnicity and preexisting health conditions. It would be interesting to see the dementia results when controlling for presence of other preexisting health conditions in addition to controlling for age/sex. I would also encourage the authors to offer some thoughts on possible mechanisms. For example, most dementia patients receive care from other people to help them with their daily activities (so require close contact with people which increases the probability of infection). And, I suggest they cite related work specifically focusing on dementia patients. For example, Wang Q.Q., Davis P.B., Gurney M.E., Xu R. "COVID-19 and dementia: Analyses of risk, disparity, and outcomes from electronic health records in the US," 2021.

4) While the authors highlight dementia and learning disability results, there is also significant evidence of an increase in racial/ethnic disparities which is consistent with other studies and I think this finding is also worth mentioning. Additionally, while small in magnitude, there is also an interesting finding that the most disadvantaged areas had a statistically significant increase in relative risk. This finding would need to be explored in future research to understand the causes behind this increase and examine whether this increase persisted as the pandemic continued.

Minor points:

1) Suggest including the entire pre-period to Figure 2 so it is easy to see how well the prediction performed.

2) Figure 3 does not include rural/urban results. Also, would it be possible to include all 5-year age categories instead of having just one 5-year age increase? In Figure 3, is everything adjusted for sex and age (including the 5-year increase in age and male categories)? 

3) I was not sure if the post-period (May 28-July 31, 2020) is included in any of the analysis. Figure 2 does not show any excess deaths in July.

4) Is the deprivation measure based on patient or practice location?

Reviewer #2: "Factors associated with excess all-cause mortality in the first wave of COVID-19 pandemic in the UK: a time-series analysis using the Clinical Practice Research Datalink" explores excess mortality in the UK from a largely-representative dataset covering some 20% of the domestic population, with a focus on the first wave of the pandemic (defined as 5th March to 27th May 2020), against expected mortality as modelled on data from 2015 onwards. The relative rate of death (RR) was computed for a large number of health and demographic factors (Figure 3), adjusted for age and sex where appropriate. An overall adjusted rate ratio of 1.43 was found for the first wave, broadly consistent with prior work on the topic, with the observation that "the net effect of COVID-19 in different sub-groups of the population is to simply amplify baseline mortality risk by a constant amount" (Line 378), though significant change in RR could be observed for some factors (e.g. dementia, learning disabilities).

The analysis is generally comprehensive and convincing. However, some points might be clarified, although they would likely not materially affect the main findings:

1. For observed vs. modelled mortality (covered in the Statistical Analysis section and Figure 2), it would be highly recommended to provide more details about the accuracy of the model in fitting known data (apparently represented as solid grey lines in Figure 2, and currently only "visually checked" from Line 143). This is because the modelled mortality underpins the entire analysis. For example, modelled vs. actual (weekly) mortality might be plotted for each of the years 2015-2019 in a (supplementary) figure, and some quantitative estimate of any deviance between modelled and actual mortality might be made, for these years.

2. Further on Figure 2, it is recommended to indicate the specific years (2015-2019) for the grey lines if possible, perhaps by using line break patterns/colours. Moreover, it appears that the predicted mortality per million (i.e. assuming no pandemic) for 2020, is lower than the previous years; is this an expected continuation of an existing pattern, and if so, what might be the cause (e.g. changing demographics due to immigration?). This might be briefly discussed.

3. While a model is required to predict the mortality for 2020 had there been no pandemic, it is not as clear as to why the model had to be fitted by splines to the observed (real) deaths in 2020 (solid red line, Line 208). Might it not be desirable to calculate excess mortality by subtracting (modelled) predicted mortality from (actual) observed mortality, instead of subtracting it from (fitted) mortality? This might be explained further, and if possible a brief sensitivity analysis on actual observed mortality might be considered.

4. The fitting for the basic generalized linear models as described in Lines 134 to 146 might be expounded upon in greater detail, possibly in supplementary material, with design choices discussed (e.g. [hyper]parameters chosen for Fourier terms/the quadratic function) with relevant references cited in support, if appropriate (there appears currently only a brief mention of a BMJ 2016 paper, in Page 17 of the supplementary material).

5. If possible, similar details might be added for the secondary analyses. For example, for the cohort approach, it is currently stated that "Interactions between the pandemic indicator and covariates will be added one at a time initially and in combinations, where possible, to describe differences in excess mortality between risk factor groups" - is there any protocol/method behind the order/combinations of such interactions?

6. The overall excess mortality found might be briefly discussed against prior works on aggregate country-level excess mortality, and not merely excess mortality by health/demographic factors; although the analysis in this manuscript involves only the population aged 40 and above, such comparisons should still be reasonable given that mortality amongst the younger population is relatively negligible.

Reviewer #3: Line 143: What do the authors mean by saying We visually checked the adequacy of our generalised linear models by comparing observed and expected deaths in the pre-pandemic period?

Is the negative binomial model an heirarchical model? If not, why not? 

Were GP practices in the study subject to data quality exclusions re reporting of outcomes and risk factors.

The observed and predicted deaths in the learning disabilities group seem to show much greater variation (in non pandemic period) than for other groups. Given that one of the conclusions of the study concerns the impact of COVID-19 on this group why did the authors not seek to diagnose miss-specification, rather than eyeball obs-v-exp? 

Line 101: Other studies have shown considerable variation in excess deaths across geographical areas much smaller than NHS Region (ref 13 in m/s). Given post-code, smaller geographical areas could have been studied, such regional variation may impact on the main findings. The authors should consider discussing this, in particular the variation across smaller geographical scales.

Also, is it not possible to resolve the urban-rural classification further, eg to include conurbation etc

Any attachments provided with reviews can be seen via the following link:

[LINK]

# Author comment: Author response to Decision Letter 1

**DOI:** 10.1371/journal.pmed.1003870.r003

4 Oct 2021

Attachment Submitted filename: Revisions letter Sept 2021 (submitted).docx Click here for additional data file.

# Peer review: Decision Letter 2

**DOI:** 10.1371/journal.pmed.1003870.r004

**Authors:** Callam Davidson

3 Nov 2021

Dear Dr. Strongman,

Thank you very much for re-submitting your manuscript "Factors associated with excess all-cause mortality in the first wave of COVID-19 pandemic in the UK: a time-series analysis using the Clinical Practice Research Datalink" (PMEDICINE-D-21-02569R2) for review by PLOS Medicine.

I have discussed the paper with my colleagues and the academic editor and it was also seen again by three reviewers. I am pleased to say that provided the remaining editorial and production issues are dealt with we are hoping to accept the paper for publication in the journal.

The remaining issues that need to be addressed are listed at the end of this email. Any accompanying reviewer attachments can be seen via the link below. Please take these into account before resubmitting your manuscript:

[LINK]

***Please note while forming your response, if your article is accepted, you may have the opportunity to make the peer review history publicly available. The record will include editor decision letters (with reviews) and your responses to reviewer comments. If eligible, we will contact you to opt in or out.***

In revising the manuscript for further consideration here, please ensure you address the specific points made by each reviewer and the editors. In your rebuttal letter you should indicate your response to the reviewers' and editors' comments and the changes you have made in the manuscript. Please submit a clean version of the paper as the main article file. A version with changes marked must also be uploaded as a marked up manuscript file.

Please also check the guidelines for revised papers at http://journals.plos.org/plosmedicine/s/revising-your-manuscript for any that apply to your paper. If you haven't already, we ask that you provide a short, non-technical Author Summary of your research to make findings accessible to a wide audience that includes both scientists and non-scientists. The Author Summary should immediately follow the Abstract in your revised manuscript. This text is subject to editorial change and should be distinct from the scientific abstract.

We hope to receive your revised manuscript within 1 week. Please email us (plosmedicine@plos.org) if you have any questions or concerns.

We ask every co-author listed on the manuscript to fill in a contributing author statement. If any of the co-authors have not filled in the statement, we will remind them to do so when the paper is revised. If all statements are not completed in a timely fashion this could hold up the re-review process. Should there be a problem getting one of your co-authors to fill in a statement we will be in contact. YOU MUST NOT ADD OR REMOVE AUTHORS UNLESS YOU HAVE ALERTED THE EDITOR HANDLING THE MANUSCRIPT TO THE CHANGE AND THEY SPECIFICALLY HAVE AGREED TO IT.

Please ensure that the paper adheres to the PLOS Data Availability Policy (see http://journals.plos.org/plosmedicine/s/data-availability), which requires that all data underlying the study's findings be provided in a repository or as Supporting Information. For data residing with a third party, authors are required to provide instructions with contact information for obtaining the data. PLOS journals do not allow statements supported by "data not shown" or "unpublished results." For such statements, authors must provide supporting data or cite public sources that include it.

To enhance the reproducibility of your results, we recommend that you deposit your laboratory protocols in protocols.io, where a protocol can be assigned its own identifier (DOI) such that it can be cited independently in the future. Additionally, PLOS ONE offers an option to publish peer-reviewed clinical study protocols. Read more information on sharing protocols at https://plos.org/protocols?utm_medium=editorial-email&utm_source=authorletters&utm_campaign=protocols

Please review your reference list to ensure that it is complete and correct. If you have cited papers that have been retracted, please include the rationale for doing so in the manuscript text, or remove these references and replace them with relevant current references. Any changes to the reference list should be mentioned in the rebuttal letter that accompanies your revised manuscript.

Please note, when your manuscript is accepted, an uncorrected proof of your manuscript will be published online ahead of the final version, unless you've already opted out via the online submission form. If, for any reason, you do not want an earlier version of your manuscript published online or are unsure if you have already indicated as such, please let the journal staff know immediately at plosmedicine@plos.org.

If you have any questions in the meantime, please contact me or the journal staff on plosmedicine@plos.org.  

We look forward to receiving the revised manuscript by Nov 10 2021 11:59PM.   

Sincerely,

Callam Davidson, 

Associate Editor 

PLOS Medicine

plosmedicine.org

------------------------------------------------------------

Requests from Editors:

Author Summary: Please trim your answer to the question 'What Did the Researchers Do and Find?' in your Author Summary, such that there are a maximum of 4 single sentence bullet points (consider combining bullets 1 and 2). 

Line 114: Please temper this claim of primacy by adding 'To our knowledge, there are no published analyses...'

Lines 128-131: Please also reference the checklist in your supplementary materials.

Line 476-477: Please remove the Ethics Approval line from your Acknowledgements section - this information was already stated in your methods.

References: In keeping with our style guidelines (https://journals.plos.org/plosmedicine/s/submission-guidelines), please add '[preprint]' after medRxiv to references 2, 8, 13, and 50.

Comments from Reviewers:

Reviewer #1: Thank you for providing answers to my comments. My initial comments were relatively minor, and I am satisfied with the answers and the changes made. I have no further comments.

Reviewer #2: We thank the authors for addressing our concerns. Do note that for the change in Line 399, "from 2015-1019" might be "from 2015-2019".

Reviewer #3: Happy with responses to review

Any attachments provided with reviews can be seen via the following link:

[LINK]

# Editor report: Decision Letter 3

**DOI:** 10.1371/journal.pmed.1003870.r005

**Authors:** Callam Davidson

17 Nov 2021

Dear Dr Strongman, 

On behalf of my colleagues and the Academic Editor, Dr Sanjay Basu, I am pleased to inform you that we have agreed to publish your manuscript "Factors associated with excess all-cause mortality in the first wave of COVID-19 pandemic in the UK: a time-series analysis using the Clinical Practice Research Datalink" (PMEDICINE-D-21-02569R3) in PLOS Medicine.

Before your manuscript can be formally accepted you will need to complete some formatting changes, which you will receive in a follow up email. Please be aware that it may take several days for you to receive this email; during this time no action is required by you. Once you have received these formatting requests, please note that your manuscript will not be scheduled for publication until you have made the required changes.

When making the formatting changes, please also make the following update:

* Line 79: update to 'can be reasonably applied in the pandemic situation, though it cannot be assumed that the observations in this study will generalise beyond Wave 1.'

In the meantime, please log into Editorial Manager at http://www.editorialmanager.com/pmedicine/, click the "Update My Information" link at the top of the page, and update your user information to ensure an efficient production process. 

PRESS

We frequently collaborate with press offices. If your institution or institutions have a press office, please notify them about your upcoming paper at this point, to enable them to help maximise its impact. If the press office is planning to promote your findings, we would be grateful if they could coordinate with medicinepress@plos.org. If you have not yet opted out of the early version process, we ask that you notify us immediately of any press plans so that we may do so on your behalf.

We also ask that you take this opportunity to read our Embargo Policy regarding the discussion, promotion and media coverage of work that is yet to be published by PLOS. As your manuscript is not yet published, it is bound by the conditions of our Embargo Policy. Please be aware that this policy is in place both to ensure that any press coverage of your article is fully substantiated and to provide a direct link between such coverage and the published work. For full details of our Embargo Policy, please visit http://www.plos.org/about/media-inquiries/embargo-policy/.

To enhance the reproducibility of your results, we recommend that you deposit your laboratory protocols in protocols.io, where a protocol can be assigned its own identifier (DOI) such that it can be cited independently in the future. Additionally, PLOS ONE offers an option to publish peer-reviewed clinical study protocols. Read more information on sharing protocols at https://plos.org/protocols?utm_medium=editorial-email&utm_source=authorletters&utm_campaign=protocols

Thank you again for submitting to PLOS Medicine. We look forward to publishing your paper. 

Sincerely, 

Callam Davidson 

Associate Editor 

PLOS Medicine
